# Chemotaxonomic and Molecular Insights into Phytoplankton Communities in Tropical Aquatic Ecosystems via MALDI FT-ICR Mass Spectrometry

**DOI:** 10.1021/acsmeasuresciau.5c00175

**Published:** 2026-03-24

**Authors:** Luis M. Díaz-Sánchez, Martha L. Aguilera, David Stranz, Scott Campbell, Luisa F. Espinosa-Díaz, Cristian Blanco-Tirado, Marianny Y. Combariza

**Affiliations:** † Escuela de Química, 28014 Universidad Industrial de Santander, Bucaramanga 680002, Colombia; ‡ Departamento de Química, 27995 Universidad de Pamplona, Pamplona 543050, Colombia; § National High Magnetic Field Laboratory, 189689 Florida State University, Tallahassee 32310, Florida, United States; ∥ Sierra Analytics, Modesto 95356, California, United States; ⊥ 120016 Instituto de Investigaciones Marinas y Costeras “José Benito Vives de Andréis”-INVEMAR, Santa Marta 470006, Colombia

**Keywords:** chemotaxonomy, phytoplankton, biomarkers, chlorophylls, cyanotoxins, MALDI, FT-ICR

## Abstract

Accurate identification of phytoplankton communities is essential for understanding the ecological dynamics of aquatic ecosystems. Conventional optical microscopy, while widely used, is labor-intensive and limited in its ability to resolve small-sized taxa or organisms present at low cell densities. Here, we present a rapid, one-step chemotaxonomic approach based on ultrahigh-resolution Matrix-Assisted Laser Desorption/Ionization Fourier Transform Ion Cyclotron Resonance Mass Spectrometry (MALDI FT-ICR MS) performed on a 21 T instrument for the molecular characterization of phytoplankton assemblages. Phytoplankton samples were collected from two contrasting sites in the Ciénaga Grande de Santa Marta (CGSM)—Ciénaga La Luna and Boca de La Barra—during the dry (June) and rainy (August) seasons of 2022. The 21 T MALDI FT-ICR MS platform enabled the simultaneous detection of chlorophylls, carotenoids, and cyanobacterial secondary metabolites directly from crude solvent extracts, generating reproducible molecular fingerprints without prior chromatographic separation. Clear spatial and seasonal differences in molecular composition were observed between sampling sites and seasons, as evidenced by distinct pigment and metabolite profiles and supported by a multivariate analysis. Specific biomarkers, including chlorophyll derivatives, diagnostic carotenoids (e.g., fucoxanthin- and zeaxanthin-related compounds), and cyanobacterial metabolites, showed qualitative agreement with phytoplankton taxa identified by optical microscopy. These results demonstrate that 21 T MALDI FT-ICR MS provides a robust and time-efficient platform for resolving chemically driven differences among phytoplankton communities and for complementing traditional taxonomic identification in complex estuarine systems.

## Graphical abstract

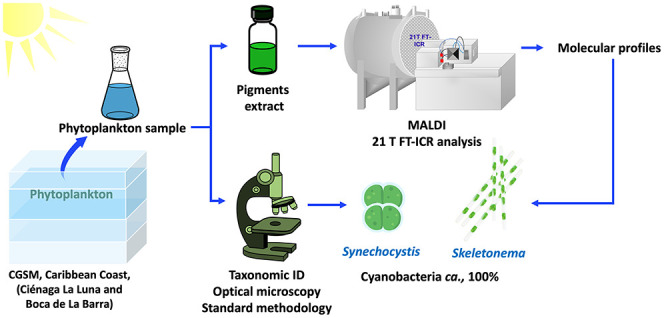

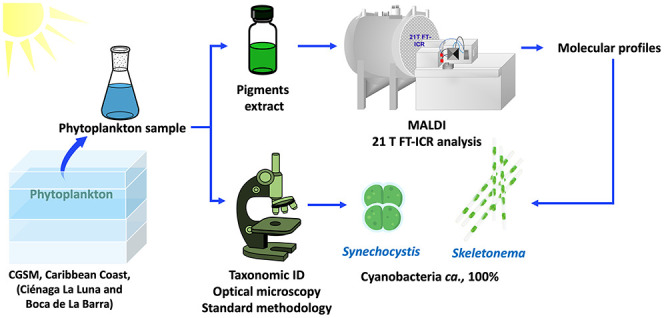

## Introduction

The phytoplankton community is a diverse group of microscopic photosynthetic organisms that includes microalgae and cyanobacteria, playing a vital role in the aquatic food chain.^1–3^ In addition to being a primary food source for many aquatic organisms, phytoplankton plays a central role in the ecosystem’s carbon and nutrient cycles. They are also prolific producers of natural organic matter, essential for the overall health and functioning of aquatic ecosystems.^4^ The ecological significance of phytoplankton extends beyond sustenance and organic matter production as contributors to oxygen production through photosynthesis and effective carbon dioxide sinks. Their photosynthetic activity plays a vital role in regulating global climate patterns and maintaining the atmospheric oxygen–carbon dioxide balance.^4,5^ Furthermore, phytoplankton contribute to various ecosystem services, including water quality regulation, nitrogen fixation, and support for higher trophic levels, making them an indispensable component of the ecosystem.

Microscopy has long been a valuable tool for identifying phytoplankton and assessing their abundance and diversity. Despite its precision, this technique has significant limitations. It is highly time-consuming, and the accuracy of species identification depends heavily on the analyst’s expertise.^6^ Furthermore, because microscopy relies solely on morphological features, it is unable to distinguish between species with similar physical characteristics.^7^ In recent years, molecular techniques, such as amplicon sequencing, shotgun sequencing, and metabarcoding, have been increasingly employed for phytoplankton identification. However, these methods are not without challenges; intragenomic variation, where multiple copies of individual genes exist, often leads to an overestimation of cell numbers and diversity. This discrepancy complicates the integration of molecular data with morphological observations. Beyond traditional morphological and molecular taxonomy, phytoplankton classification has also considered functional, ecometabolomic, and chemotaxonomic traits.^8,9^ These approaches not only enhance taxonomic resolution but also provide valuable insights for ecological research.^3,10^

A variety of analytical techniques have been employed for the chemical and biological characterization of phytoplankton and other aquatic biota. Spectrophotometric pigment analysis and fluorometric approaches are widely used for estimating biomass and for distinguishing broad functional groups based on chlorophylls and accessory pigments.^5,11–14^ Similarly, flow cytometry coupled with fluorescence detection enables rapid assessment of cell abundance and size distributions,^15^ while advanced fluorescence and optical microscopy techniques provide valuable morphological and physiological information.^16^ Nuclear magnetic resonance (NMR) spectroscopy has also been applied for metabolomic studies of aquatic organisms, offering structural insights into major metabolite classes.^17,18^

Despite their utility, these approaches generally lack the molecular specificity required to unambiguously differentiate biomarker phytoplankton species or closely related taxa, particularly in complex natural assemblages. Pigment-based UV–vis and fluorescence methods, for instance, often suffer from spectral overlap among pigments shared across multiple taxa, limiting their taxonomic resolution.^2,19–22^ These limitations have been extensively discussed in the literature and underscore the need for high-resolution molecular-level techniques capable of resolving complex mixtures of phytoplankton biomarkers.

However, molecular-level characterization of phytoplankton biomarkers poses analytical challenges due to their chemical complexity and the presence of high molecular weight and/or heteroatom-bearing species, such as carbohydrates, lignocellulosic compounds, pigments, lipids, and many other yet-to-be-characterized compounds.^23–25^ Pigment analyses in phytoplankton samples typically involve specific and laborious protocols, often including fractionation steps and the use of various complementary instruments, all aimed at enhancing sensitivity, resolution, and species identification. Recently, we have reported an electron-transfer Matrix-Assisted Laser Desorption/Ionization Mass Spectrometry (MALDI MS) methodology for the characterization of microalgae pigments, yielding results comparable to, or even surpassing, those achieved with standard techniques such as High-Performance Liquid Chromatography coupled to UV–vis detection (HPLC–UV/vis).^26^ MALDI MS offers several advantages for generating phytoplankton pigment profiles, including ionization selectivity, molecular ion survival, high impurity tolerance, and low detection limits.^27^

In positive ion mode, the ET mechanism involves electron transfer from an analyte (A) neutral molecule with ionization energy (*Ei*) below the matrix’s *Ei* to a primary ion of the matrix (M). This process results in the formation of an analyte molecular ion (A^+•^)^28–33^ according to the following reaction pathway:

$$M^{+\cdot }+A\to A^{+\cdot }+M$$

$$E_{i\left(Matrix\right)}>E_{i\left(Analyte\right)}∴{\Delta E}_{i}>0.5eV$$

Previous work shows that ET MALDI MS selectively ionizes chlorophylls, carotenoids, and lipids, identifying low-abundance biomarkers crucial for phytoplankton studies.^26,34,35^ MALDI Fourier Transform Ion Cyclotron Resonance (MALDI FT-ICR) stands out as a powerful technique for targeted analysis of biomarkers in complex mixtures. Recent advancements in single-run methods enable the simultaneous monitoring of multiple target biomarkers, utilizing the capability of FT-ICR MS to screen for thousands of compounds with high resolution over a broad analytical window of polarity and molecular weights.^36–40^ Furthermore, MALDI FT-ICR MS approaches simplify sample preparation and have broad-range screening capabilities that can reveal new biomarkers. In addition, the detection of fine isotopic patterns enables the unequivocal identification of molecular species. Analysis using high-resolution mass spectrometry provides valuable compositional information that can guide subsequent confirmatory studies using targeted approaches. Previous work has demonstrated the utility of FT-ICR MS methods for detecting and characterizing diverse pigment species, including chlorophyll and carotenoid derivatives, based on accurate mass measurements and high-resolution molecular profiling, often aided by Kendrick mass defect plots and related visualization strategies.^36,41,42^

In this context, we employed our recently reported ET MALDI FT-ICR methodology^26,34^ to evaluate the molecular composition of phytoplankton samples collected at two distinct points at the Ciénaga Grande de Santa Marta (CGSM), during different seasons. The CGSM, Colombia’s largest coastal wetland, spans 4,280 km^2^ between the Caribbean Sea and Santa Marta Mountains.^43^ Renowned for its biodiversity, this ecosystem includes lagoons, estuaries, marshes, and mangroves, supporting diverse habitats.^44^ It holds five conservation designations, including Ramsar Wetland and Biosphere Reserve.^45^ As a biogeochemical interface, CGSM estuaries link land and sea, with river discharges introducing organic matter that shapes salinity and chemical gradients.^46^ CGSM also faces significant anthropogenic impacts that threaten its biodiversity and have caused loss of mangrove forests and death of fish populations.^44^ These dynamic conditions drive microbial community structures, influencing the composition of water and sediments.

The objective of this study was to investigate the molecular composition of phytoplankton samples in freshwater and Caribbean-connected environments using a chemotaxonomic approach based on ultrahigh-resolution MALDI FT-ICR MS, in order to gain fresh insights into the intricate chemistry of this estuarine system. This comprehensive analysis has the potential to elucidate the complex network of interactions within the CGSM, shedding light on the influence of anthropogenic activities and environmental factors on phytoplankton communities and the wider ecosystem.

## Methods

### Study Area

The CGSM encompasses a vast estuarine complex situated in the northern coastal region of Colombia (Department of Magdalena), between 10°44′ and 11°00′ N latitude and 74°16′ and 74°31′ W longitude. Isolated from the Caribbean Sea by Isla de Salamanca, the CGSM is bordered by the floodplain of the Magdalena River to the west and southwest and the Sierra Nevada de Santa Marta (SNSM) to the east and southeast (Figure 1). The marsh covers an area of 4,280 km^2^, with a depth ranging from 1.8 to 3 m.^46^ The CGSM’s hydrological dynamics are governed by freshwater inflows from the Magdalena River and several rivers descending from the Sierra Nevada de Santa Marta, such as the Fundación, Aracataca, and Sevilla, among others, as well as saltwater from the Caribbean Sea. Water exchange is facilitated through a natural opening in the northeast corner of the marsh. The eastern and southeastern sectors are affected by tributaries from the SNSM that provide consistent water flow. In contrast, the western and northwestern sectors are directly influenced by the Magdalena River watershed where water flow depends on rainfall.^45^ Seasonal and regional fluctuations in salinity range from 0 to 40, while water temperatures maintain an average of 30 °C annually. Global climatic phenomena, such as El Niño and La Niña, have a partial impact on water resource inputs, leading to modifications in tributary discharge and salinity parameters, thereby altering the water quality of CGSM.^45^ Despite these influences, the CGSM demonstrates high productivity, as evidenced by its notably elevated annual gross primary production rates.^45^

**Figure 1:**
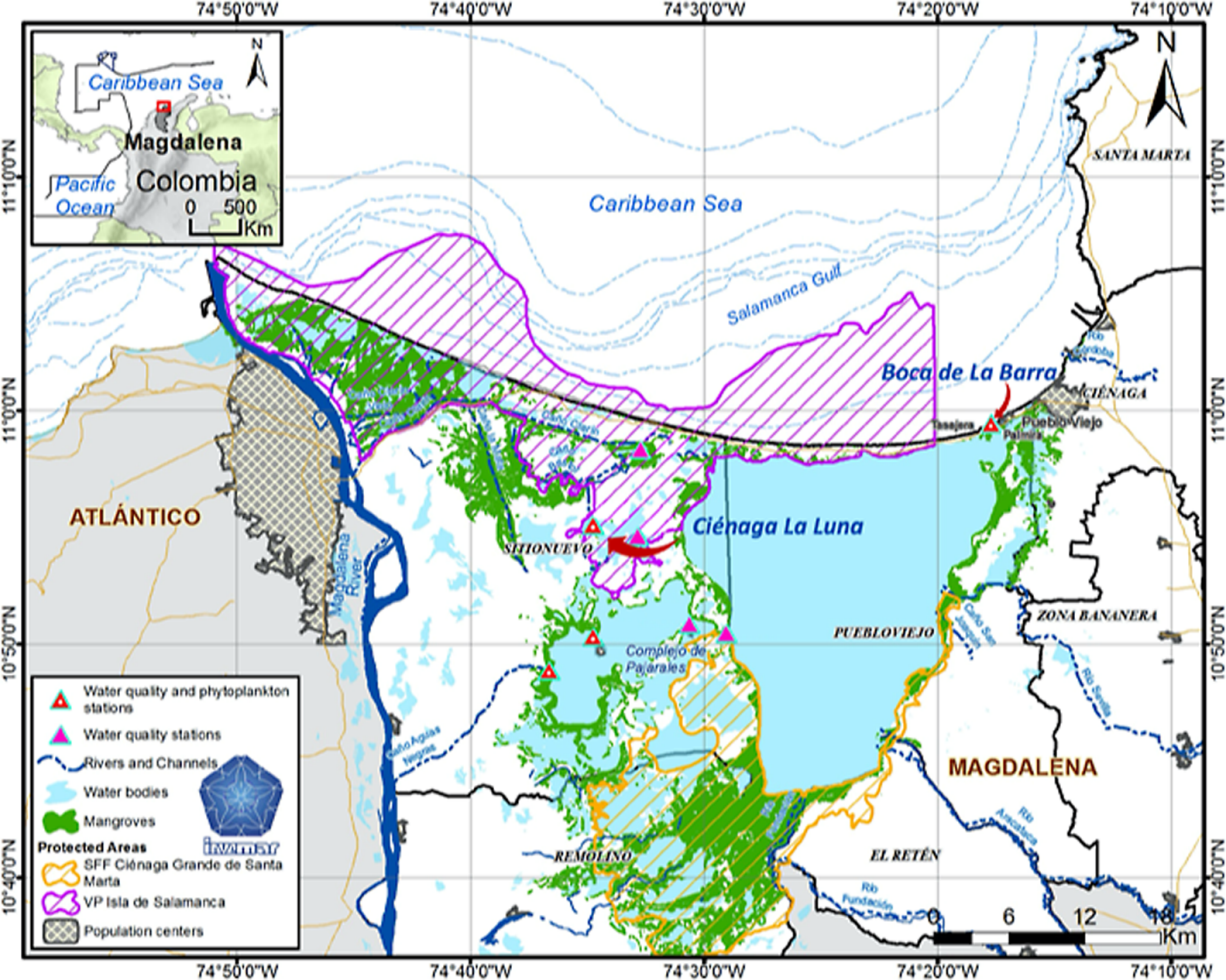
Map of the Ciénaga Grande de Santa Marta (CGSM) in the Colombian Caribbean Sea, showing the locations where phytoplankton samples were collected, along with additional sampling points designated by INVEMAR for water quality and phytoplankton monitoring. The map also depicts rivers, channels, water bodies, mangroves, and protected areas comprising the CGSM. Adapted with permission from.^47^

### Water and Phytoplankton Sampling

The samples were collected at two stations: Boca de La Barra (located between 10°59′38.47” and 74°17′39.63”) and Ciénaga La Luna (located between 10°55′7.31” and 74°34′45.56”). These stations, established by the Instituto de Investigaciones Marinas y Costeras José Benito Vives de Andréis (INVEMAR) are part of a network of 28 sampling stations situated across various zones within the CGSM complex, each exhibiting different hydrographic characteristics.^45^ Water samples were collected in June and August of 2022, corresponding to dry and rainy seasons, respectively, between 07:00 and 11:00 h (Figure 1). Boca de La Barra is a key sampling place where the CGSM estuarine complex and the Caribbean Sea connect, while Ciénaga La Luna is where the CGSM is most influenced by the Magdalena River and the floodplain.

Phytoplankton samples were provided by the INVEMAR technical team. The samples were collected using a standard conical phytoplankton net (30 cm diameter, 70 cm length) fitted with a metal bridle ring, a 23 μm polyester mesh net, a cod end PVC collection cup (250 mL) with two side windows covered with the same mesh as the net, and a rope (Biológika, Medellín, Colombia).^45^ The net was deployed at the sampling point and continuously towed at low speed for 5 min, following a prior rinsing of both the net and the cod end. The samples were transferred to amber-colored bottles and stored in an ice bath for approximately 2 h until they reached the laboratory. The samples were then filtered using Whatman 0.7 μm glass fiber filters (GF/F) and stored for pigment extraction and analysis. Additionally, for phytoplankton community taxonomy, water samples were collected following the manual and guides of the intergovernmental Oceanographic Commission, UNESCO.^48^ The samples were deposited in 250 mL polyethylene bottles, preserved with Lugol’s iodine solution at a ratio of 1:100,^49^ and stored away from direct sunlight until transportation to the laboratory for analysis. The overall experimental workflow, including phytoplankton collection, sample partitioning for taxonomic identification and molecular analysis, and subsequent processing steps, is summarized in the Supporting Information (Figure S1).

### Water Physicochemical Parameters Measurement

For the measurement of environmental and physicochemical parameters, water samples were collected in biological triplicate at each site and during each season. These triplicate samples were processed and analyzed independently, and the resulting data were used to characterize the environmental context associated with the phytoplankton samples (Supporting Information Table S1). *In situ* water salinity, temperature, and pH measurements were performed by the INVEMAR technical team using multiparameter hand-held meters HI 98194 (HANNA instruments, Woonsocket, Rhode Island (RI), USA) following Standard Methods 2520-B (salinity, temperature) and Standard Methods No. 4500-H B (pH). *In situ* dissolved oxygen (DO) was measured with a membrane electrode according to Standard Methods 4500-O G. Transparency was evaluated using a Secchi disk.^45^ Values for chemical environmental factors, such as orthophosphate (P-PO_4_), which is related to excess nutrients from anthropogenic sources, were determined using the ascorbic acid colorimetric method as previously described.^45^

### Phytoplankton Community Structure Analysis

The phytoplankton taxonomic analysis follows a series of standard processes. Initially, the Utermöhl sedimentation method was employed. Settled phytoplankton cells, previously placed into a settling chamber, were examined using an inverted microscope.^49^ Genus-level identification was conducted based on morphological cell characteristics. This involves the use of taxonomic descriptions, such as cell shape, size, the presence of specific organelles, colony formation, and keys from the scientific literature.^16,50–52^ Qualitative and quantitative information about phytoplankton was organized into matrices for calculating relative abundances per taxonomic group and for generating graphs to determine the overall behavior of the communities.

### Phytoplankton Chemical Profiles

#### Pigment Extraction

The extraction of pigments from the phytoplankton samples followed established procedures.^26,34^ One-quarter piece of the filter with the retained phytoplankton was placed in contact with 1 mL of analytical-grade acetone in a 1.5 mL amber vial. The mixture was then sonicated at 40 kHz in a Branson UltrasonicsTM CPX bath (Danbury, CT, USA), operating at 35 W for 25 min at room temperature. Following the extraction period, the sample was filtered using 0.45 mm Whatman GF/F filters, completely dried under a gentle argon stream, and stored in amber vials at 4 °C until analysis. Chlorophyll *a* quantitation was carried out by UV–vis spectroscopy using the same dried extracts according to the Lorenzen method as described in Standard Methods 10200-H.^53^

### MALDI FT-ICR MS Pigment Analysis

A 5 mM solution of *trans*-2-[3-(4-*tert*-butylphenyl)-2-methyl-2-propenylidene] malononitrile (DCTB) in acetonitrile (ACN) was prepared by dissolving the solid with the aid of ultrasound energy (40 kHz) for 2 min. Phytoplankton extracts were obtained as described above, and after solvent removal, the dried extracts were redissolved in acetonitrile (ACN) prior to MALDI FT-ICR MS analysis, matching the solvent used for the MALDI matrix to ensure homogeneous matrix–analyte mixing. The concentration of the phytoplankton extracts was estimated to be approximately 0.03 mM, based on the concentration of chlorophyll *a*, a widely accepted proxy for total phytoplankton biomass and extract content because it is the primary photosynthetic pigment present in all major phytoplankton groups.^45,53,54^ DCTB and phytoplankton solutions were mixed to reach an analyte-to-matrix ratio of 1:100. Samples (1 μL) were dispensed onto a stainless-steel sample holder by using the dried droplet method. LDI experiments were performed as a blank using DCTB under identical MALDI conditions to identify the characteristic matrix-related ions. MALDI FT-ICR experiments were carried out by using a 21 T FT-ICR mass spectrometer at the National High Magnetic Field Laboratory (NHMFL), Florida State University. The instrumental setup comprises a Velos Pro linear ion trap (Thermo Scientific, San Jose, CA) front end along with a proprietary NHMFL ICR cell and ion transfer optics. The dynamically harmonized ICR cell operated at a trapping potential of 7.5 V. Ionization was performed at an elevated-pressure MALDI source that included a dual-ion funnel interface (Spectroglyph LLC, Kennewick, WA). The funnels voltages were set at 625 kHz with a 150 *V*_p‑p_ for the first high-pressure ion funnel and 1.2 MHz with 90 *V*_p‑p_ for the second low-pressure ion funnel. An electric field gradient of approximately 10 V/cm was maintained within the dual-funnel system with a gradient of 100 V/cm between the source and the funnel inlet. The MALDI source was fitted with a Q-switched, frequency-tripled Nd:YLF laser emitting 349 nm photons (Explorer One, Spectra Physics, Mountain View, CA). The laser operated at a repetition rate of 1 kHz with a pulse energy of approximately 1.2 μJ. During the mass spectrometry analysis, an ion injection time of 250 ms was used with automatic gain control (AGC) turned off. For ultrahigh mass resolving power analyses, a transient duration of 3.1 s was employed. All spectra were obtained in positive mode. The time-domain transients were acquired using the Predator data station with an average of 100 time-domain acquisitions for all experiments.

### Elemental Composition Assignment and Data Processing

After data collection, data processing and visualization were conducted using the Investigator software (v. 1.3, Sierra Analytics, Modesto, CA) and Origin Pro 9.0 (64 bit), following the manufacturer’s guidelines. Prior to sample analysis, the mass spectrometer was externally calibrated using a Pierce LTQ Velos ESI Positive Ion Calibration Solution (Thermo Scientific), consisting of a peptide mixture covering the relevant *m*/*z* range. After data acquisition, the mass spectra were internally recalibrated using a series of highly abundant known ions, and the recalibration equations were provided by the Investigator software.

Elemental composition assignment was performed de novo by exploiting the ultrahigh mass accuracy and resolving power of the 21 T MALDI FT-ICR MS system. Monoisotopic peak detection and isotope cluster recognition were carried out automatically with Investigator software. Molecular formulas containing C, H, O, N, and S were generated under predefined elemental constraints (C_1–60_, H_1–100_, N_0–4_, O_0–15_, S_0–2_) with a mass error tolerance of ≤250 ppb.^40^ Additional chemical constraints included a maximum hydrogen-to-carbon (H/C) ratio of 2 and a double bond equivalent (DBE) limit of 30.

Only singly charged molecular ions ([M]^+•^, [M + H]^+^, and [M + Na]^+^) with mass errors below 250 ppb^40^ and relative abundances above 0.2%^39^ were retained for further analysis, ensuring unambiguous molecular formula assignments while minimizing the contribution of spurious signals. Molecular formula assignments were further validated by comparing experimental isotopic patterns with theoretical distributions calculated using ChemCalc.^55^ A resolving power of approximately 1.6 × 10^6^ at *m*/*z* 400 was achieved for all spectra.

Molecular descriptors, including DBE, H/C, and O/C ratios, were calculated automatically by the Investigator software for each assigned molecular formula. Relative abundances (RA) were generated independently for each mass spectrum by normalizing the intensity of each assigned ion to the total ion intensity within the same spectrum. No external normalization or imputation of missing values was applied as the analysis was focused on qualitative molecular fingerprinting. Carotenoids and cyanobacteria’s secondary metabolites were tentatively identified by cross-referencing the list of molecular formulas assigned with the Investigator software with the Carotenoids Database^56^ and CyanoMetDBm,^57^ respectively.

## Results and Discussion

### Phytoplankton Communities in the CGSM

In aquatic ecosystems, the composition and density of phytoplankton communities are closely linked to the water quality parameters. Environmental parameters reported by the INVEMAR at the sampling points Ciénaga La Luna and Boca de La Barra in June and August of 2022 are in the Supporting Information, Table S1. Microscopic analysis of phytoplankton samples collected at Ciénaga La Luna and Boca de La Barra identified five taxonomic groups (phyla), with Cyanophyta (commonly known as cyanobacteria) being the most prevalent (Figure 2).

**Figure 2:**
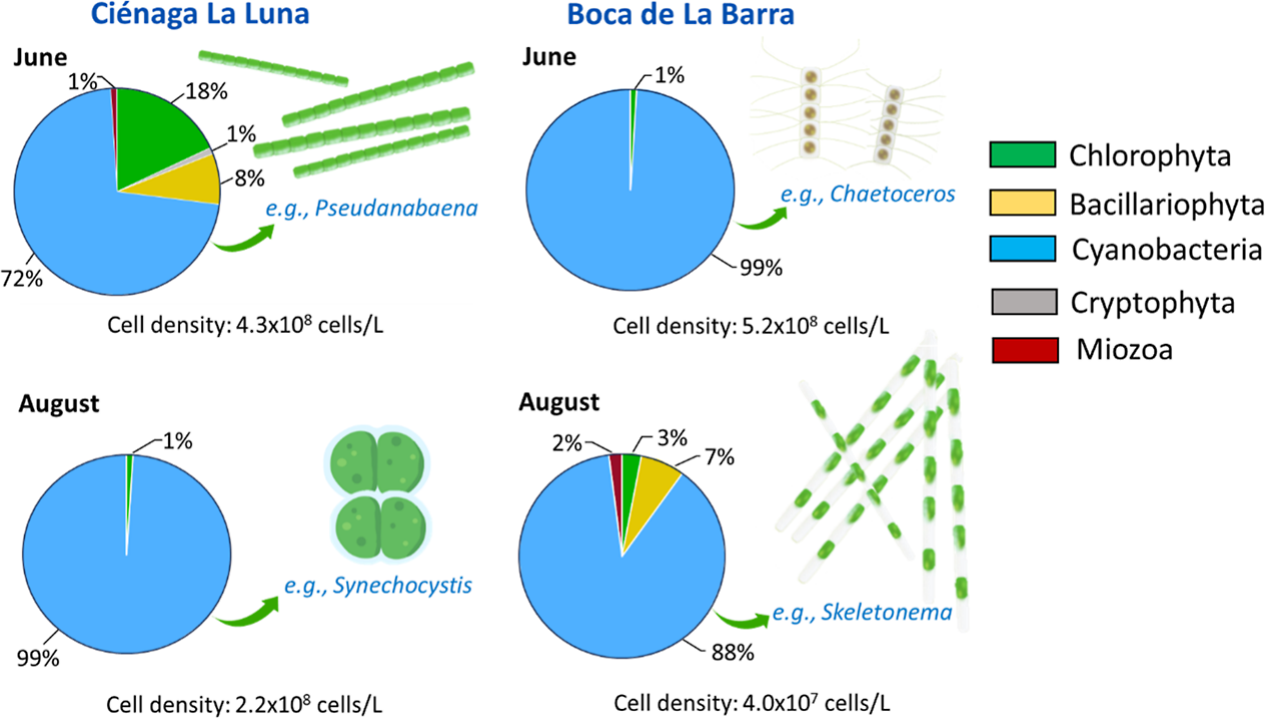
Relative abundance of the main taxonomic groups of phytoplankton at the sampling points Ciénaga La Luna and Boca de La Barra, during June and August of 2022, along with cell density counts. Insets illustrate some of the most abundant potentially harmful microalgae species (dominant cyanobacterial taxa) identified at each sampling point.

According to Figure 2, the phytoplankton cell density decreased significantly for the month of August, the high precipitation season, compared to June, the low precipitation season, in both sampling stations. Furthermore, cellular density values are closely related to phytoplankton concentration. These results fall within ranges previously reported by the INVEMAR during monitoring conducted from 2014 to 2021.^45^ The data from this work show that the maximum value of total phytoplankton biomass occurs during the dry season, which is consistent with reports in scientific literature.^43^ On average, the highest phytoplankton densities were found in Ciénaga La Luna, the sampling point with the most significant influence of continental water inputs. Additionally, according to the literature, higher salinity values typically favor greater phytoplankton densities.^58,59^ In this study, a low diversity of microorganisms was detected in the phytoplankton samples, a finding also reported by other authors.^45,60^ The authors suggest that aquatic ecosystems in a eutrophic state are characterized by low phytoplankton diversity and the predominance of particular species or groups, as observed in CGSM with cyanobacteria. Systems exhibiting a decrease in microalgae richness may be more susceptible to intense or prolonged environmental changes.^45^

### Pigment Profile Analysis by MALDI FT-ICR

The pigment profiles of phytoplankton extracts collected from Ciénaga La Luna and Boca de La Barra in June and August 2022, analyzed using a 21 T FT-ICR mass spectrometer, are depicted in Figure 3. Postacquisition data processing was performed using Investigator (version 1.3, Sierra Analytics, Modesto, CA). Monoisotopic ions were identified from isotope clusters, generating charge-deconvoluted neutral-mass spectra. Mass difference tolerances were iteratively optimized (1 mDa), with 0.2 mDa for isotope peak spacing.^61^ Elemental composition assignment was performed de novo, applying defined parameters (see the Methods section). After the data filtration, 11,108 molecular formulas were assigned from a total of 11,417 monoisotopic signals detected with relative abundances (RA) above 0.2% in June, Boca de La Barra. Likewise, in the phytoplankton samples collected in Ciénaga La Luna, June, we assigned 2,808 molecular formulas from a total of 2,995 monoisotopic signals detected above 0.2% RA. The analysis was conducted for signals within the mass range of *m*/*z* 200–1200, with peak resolving power exceeding 1.6 × 10^6^ at *m*/*z* 400. Mass accuracy measurements were within the range of 1 to 250 ppb.^40^ The presented results address only the extractable and ionizable component in the phytoplankton samples. The mass spectrum provided a high-resolution view of the isotope fine structure of the peaks, closely aligned with spectra calculated for the same resolving power using the ChemCal molecular formula calculator algorithm.^55^

**Figure 3:**
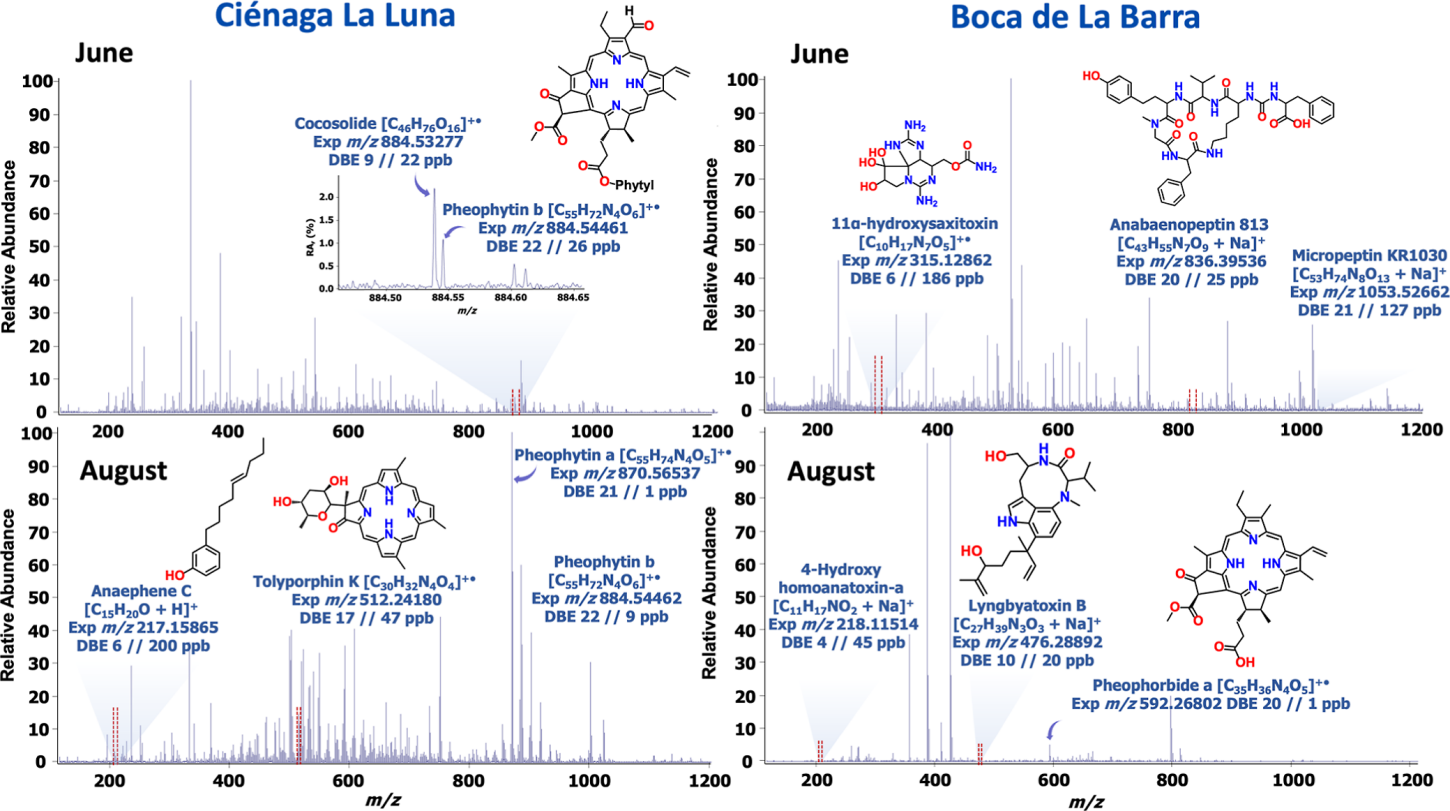
MALDI FT-ICR MS spectra of phytoplankton samples collected in June and August 2022 from Ciénaga La Luna and Boca de La Barra, CGSM. The resolving power at *m*/Δ*m*_50%_ is ca., 1.6 × 10^6^ (*m*/*z* 400). Insets offer details on the molecular formula, experimental mass, mass accuracy, and double bond equivalent (DBE) for representative compounds identified based on ultrahigh-resolution accurate mass measurements and de novo elemental composition assignment. Some assigned molecular formulas are not associated with a unique molecular structure within the scope of this study.

Figure 3 shows the diverse molecular composition observed among the samples collected in June and August 2022 at Ciénaga La Luna and Boca de La Barra, CGSM. We detected chlorophylls and their derivatives, carotenoids, and secondary metabolites produced by cyanobacteria, detected in part as radical cation species, protonated molecules, and sodium adducts. The chlorophyll derivatives were identified solely as radical cations due to successful charge-transfer reactions between the matrix primary ions (DCTB, *E*_i_: 8.54 eV) and the chlorine-based structures with characteristic low ionization energy (*E*_i_) values.^26,34,35^ Carotenoids and secondary metabolites of cyanobacteria were detected as [M]^+•^, [M + H]^+^, or [M + Na]^+^ depending upon the molecule’s structure, *E*_i_, and cation/proton affinities, as discussed later. More compounds were identified in June than in August at both sampling sites, Ciénaga La Luna and Boca de La Barra. This correlates well with the cell density values recorded in June (4.3 × 10^8^ and 5.2 × 10^8^ cells/L) and August (2.2 × 10^8^ and 4.0 × 10^7^ cells/L) for Ciénaga La Luna and Boca de La Barra, respectively. The variation in molecular composition can be attributed to various environmental and temporal factors that affect phytoplankton communities in the CGSM. To observe the ionization behavior across compound classes, it is necessary to first outline the overall molecular diversity present in the phytoplankton samples. This provides a more comprehensive framework for understanding the ion types generated under the MALDI FT-ICR MS conditions. Some assigned molecular formulas may represent constitutional isomers that cannot be resolved with the present approach. Future work incorporating ion mobility spectrometry (IMS) could enable the improved differentiation of structural variants within complex phytoplankton extracts.

Figure 4 presents the compositional information derived from MALDI FT-ICR MS in the form of compound class histograms. Each class is divided into three sections, showing the percentage of monoisotopic ions detected as radical cations ([M]^+•^), protonated molecules ([M + H]^+^), and sodium adducts ([M + Na]^+^). A wide diversity of compound classes was detected, with Table S2 compiling the compositional information derived from MALDI FT-ICR MS for the compound classes identified in phytoplankton samples.

**Figure 4:**
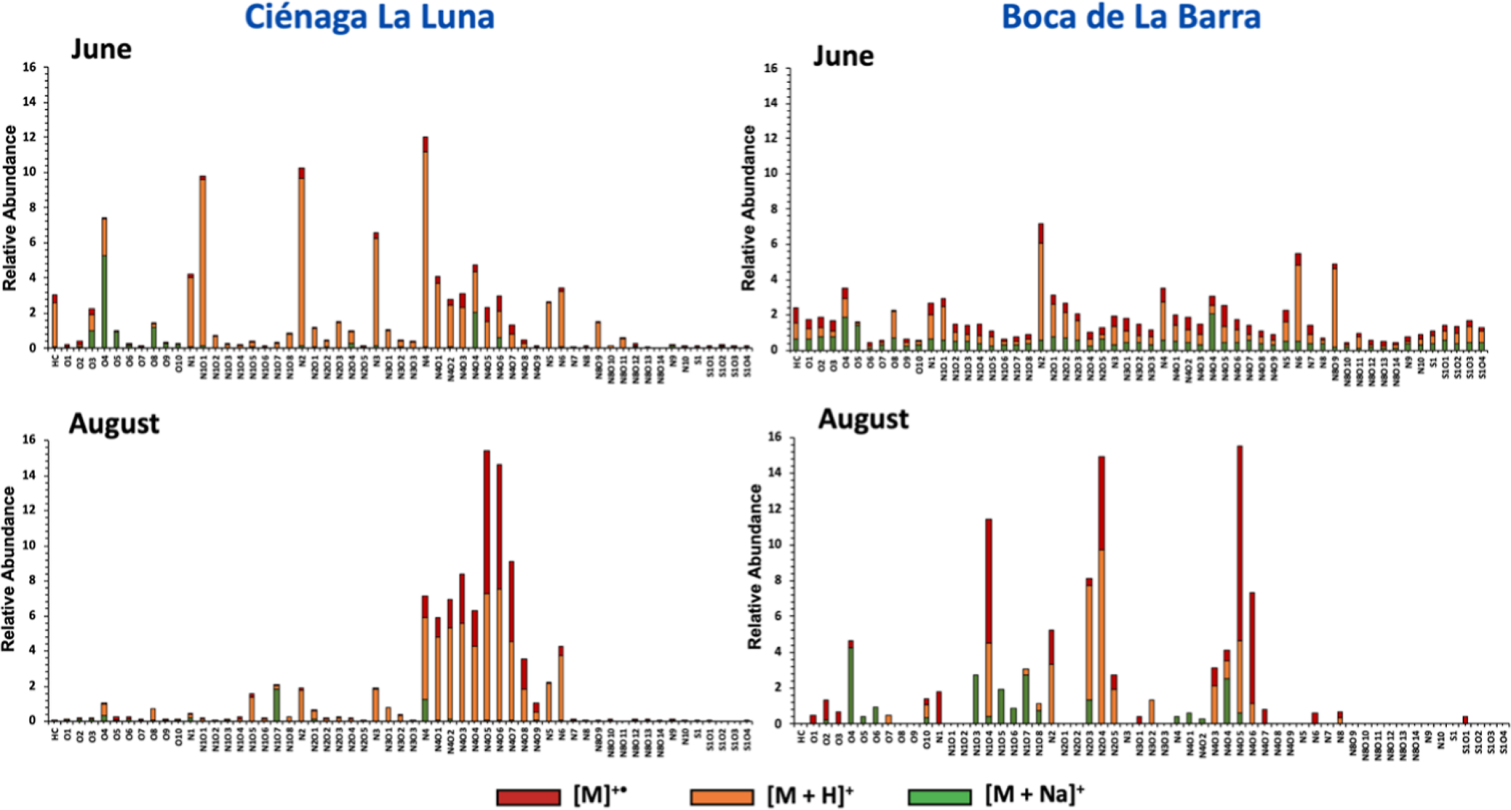
Comparative histograms showing compound classes detected in phytoplankton samples collected in June and August at Ciénaga La Luna and Boca de la Barra, CGSM. Each class is divided into three sections showing the percentage of monoisotopic ions detected as radical cations ([M]^+•^) in the red section, protonated molecules ([M + H]^+^) in the orange section, and sodium adducts ([M + Na]^+^) in the green section. Assigned molecular formulas indicate high-confidence elemental assignments; however, not all are connected to a specific molecular structure within this study’s scope.

Figure 4 shows the relative abundances of the different detected species ([M]^+•^, [M + H]^+^, [M + Na]^+^) providing information about the ionization process, which can occur through different pathways using an ET MALDI matrix such as DCTB. Although radical cations were detected for almost all compound classes, protonation was the ionization pathway with the highest relative abundance. Some authors, including our previous studies,^26^ have reported that DCTB can induce protonation of other molecules in MALDI ionization due to the presence of an acidic hydrogen in its structure. For this ionization to occur, the proton affinity of the analyte must be greater than the proton affinity of DCTB.^29,62–64^ Compounds detected as [M + H]^+^ or [M + Na]^+^ (see Table S2) are compounds with high nitrogen or oxygen content, conferring high proton and cation affinities. Considering the origin of the analyzed samples, with high salt concentrations and minimal pretreatment before analysis, the presence of pre-existing ionic species in solution, requiring only MALDI matrix assistance for desorption in the MALDI ionization chamber, is possible. N_4_O_o_ (o = 1, 2, 3, 4, 5, 6, 7, 8, 9) were the predominant species detected as radical cations, possibly because they are related to chlorin derivatives, with *E*_i_ lower than the *E*_i_ of the MALDI matrix DCTB (*E*_i_: 8.54 eV). In August, the highest percentage of radical cations was detected in both Ciénaga La Luna and Boca de La Barra, possibly due to increased precipitation, reducing dissolved salt concentration and other nutrients on the water surface.

A total of 57 compound families were detected, of which 32 were common in both June and August at Ciénaga La Luna and Boca de Barra, e.g., O_1_, O_2_, O_3_, O_4_, O_5_, O_6_, O_7_, N_1_, N_1_O_3_, N_1_O_4_, N_1_O_5_, N_1_O_6_, N_1_O_7_, N_1_O_8_, N_2_, N_2_O_3_, N_2_O_4_, N_2_O_5_, N_4_, N_4_O_1_, N_4_O_2_, N_4_O_3_, N_4_O_4_, N_4_O_5_, N_4_O_6_, N_4_O_7_, N_6_, N_8_. These compound classes have been previously reported by other authors in analyses of Natural Organic Matter (NOM) from rivers and oceans.^65,66^ Microbial degradation is one of the most important processes in regulating the NOM composition in aquatic environments. Microorganisms are consistently involved in both supplying and depleting NOM, derived from detritus and secretions of aquatic microorganisms. Our observation of highly oxygenated compound classes O_o_ (o = 1, 2, 3, 4, 5, 6, 7, 8, 9, 10) in phytoplankton samples aligns with previous reports where O_o_ (o = 1, 2, 3, 4, 5, 6, 7) were detected in analyses of organic matter composition in rivers using APPI FT-ICR MS.^65^ Within the oxygen-containing compound classes, O_4_ was the most abundantly detected class in all samples, containing compounds such as puna’auic acid (C_18_H_32_O_4_) and 15,16-dihydrosacrolide A (C_18_H_30_O_4_), identified as [M + Na]^+^ and [M + H]^+^, respectively.

In other reports, species of class O_2_ in negative ion mode analysis are typically assigned as naphthenic acids in samples from biodegraded petroleum reservoirs. However, petrogenic naphthenic acids exhibit a uniform carbon distribution, characteristic of the geochemical signatures of fossil organic matter formed via catagenetic processes occurring over geological time scales.^67^ In contrast, the species of class O_2_ detected in positive ion mode analysis in this work exhibit short carbon distributions characteristic of natural organic matter, as previously reported by other authors.^68^ Some authors have also reported class O_2_ species with short carbon distributions in recently deposited organic matter in modern sedimentary environments.^69^ Detected O_2_ species are related to carotenoids, including compounds such as alloxanthin (C_40_H_52_O_2_) and zeaxanthin (C_40_H_56_O_2_), 4-oxo-beta-apo-13-carotenone (C_18_H_24_O_2_), all detected as [M]^+•^. The latter, a marker of the cyanobacterium Anabaena, was also reported through taxonomic ID. Additionally, toxins belonging to class O_2_ were identified, e.g., 3-oxo-*b*-ionone (C_13_H_18_O_2_), cyclic peptides previously reported in cyanobacteria *Nostoc commune*.^57^ Furthermore, other authors have linked these oxygenated classes to long-chain diketones, i.e., diones, analogous to well-known alkynones and related to species C_31_, C_33_, and C_35_, with low DBE values. Several authors^65,70,71^ have reported the degradative oxidation of other organic species, both xenobiotics such as polycyclic aromatic hydrocarbons, and biogenic ones, such as triterpenoids, to their analogues.

Among nitrogen-containing compound classes, N_4_O_o_ (o = 0, 1, 2, 3, 4, 5, 6, 7, 8, 9) were the most abundant in all samples, associated with tetrapyrrole derivatives (i.e., porphyrins) from chlorophyll pigments, as reported in previous studies by us and other authors.^65^ These molecular characteristics have been extensively studied and reported in numerous FTI-CR MS investigations of petroleum porphyrins, the end products of the geochemical transformation of biogenic chlorophyll over geological time scales.^67,72,73^ Moreover, other studies corroborate N_4_O_o_ species as representatives of primary producers in bacterial communities, linking the influx of phototroph-derived organic matter to sediments in high-salinity sites with increased abundance of specific Proteobacteria taxa efficient in consuming sedimented phototroph biomass.^74^ The authors suggest that the detection of N_4_O_3_ species is an indicator of the transition to a marine-like environment from a freshwater-influenced environment within the estuary. Furthermore, classes like N_1_O_o_ (o = 2, 3, 4, 5, 6) have been reported in marine sediments and associated with the presence of structural sphingolipids from marine phytoplankton.^65^ Sphingolipids constitute a chemically and functionally diverse group of membrane lipids, many of which include N_1_O_o_ linked to long alkyl chains. In our work, the N_1_O_o_ species were mostly detected as [M + H]^+^ or [M + Na]^+^. High abundances of N_1_O_o_ species in sediment samples have been associated with increased bacterial activities at the water–sediment interface. Additionally, NOM components containing nitrogen stabilize in the water column through interactions with minerals.^75^

In contrast, classes like S_1_O_o_ (o = 2, 3, 4) were primarily detected in June compared to August at both sampling points. Sulfur species were detected as radical cations, protonated molecules, and sodium adducts. Previous studies have reported the presence of these species in FT-ICR MS analyses of river samples.^65,76,77^ The increase in relative abundance of S_1_O_o_ classes from June to August at both sampling points may be related to increased precipitation in August in the CGSM. Sulfur compounds may originate from anthropogenic activities near CGSM or algae production (containing sulfur-containing cysteine, methionine) and may also result from early diagenetic sulfurization of DOM.^76,77^ The presence of sulfur compounds has been associated with the presence of thiophene compounds. Although the detection of thiophene compounds has not been previously reported in Ciénaga La Luna and Boca de La Barra, there are numerous reports in the literature showing anthropogenic interventions since the 1950s in CGSM^16,43,46,47^ which may be related to the addition of polycyclic aromatic hydrocarbons and, potentially, thiophene-related compounds. Previous studies on biodegraded crude oils have interpreted that the S_1_O_2_ compound class consists of acidic species, likely containing a sulfur and carboxyl functional group or a sulfur and two hydroxyl functional groups, which are degradation products of original species found in the sulfur (S) compound class.^25^ Additionally, compositional differences at the sampling points may be related to differences in salinity, as previously reported by other authors.^46,78^

The molecular composition of the phytoplankton extracts is composed of chlorophylls and their derivatives, carotenoids, and secondary cyanobacteria metabolites (cyanotoxins). Figure 5 shows the chlorophyll derivatives and carotenoids detected in the samples collected in June and August 2022 at Ciénaga La Luna and Boca de La Barra, CGSM by MALDI FT-ICR MS.

**Figure 5:**
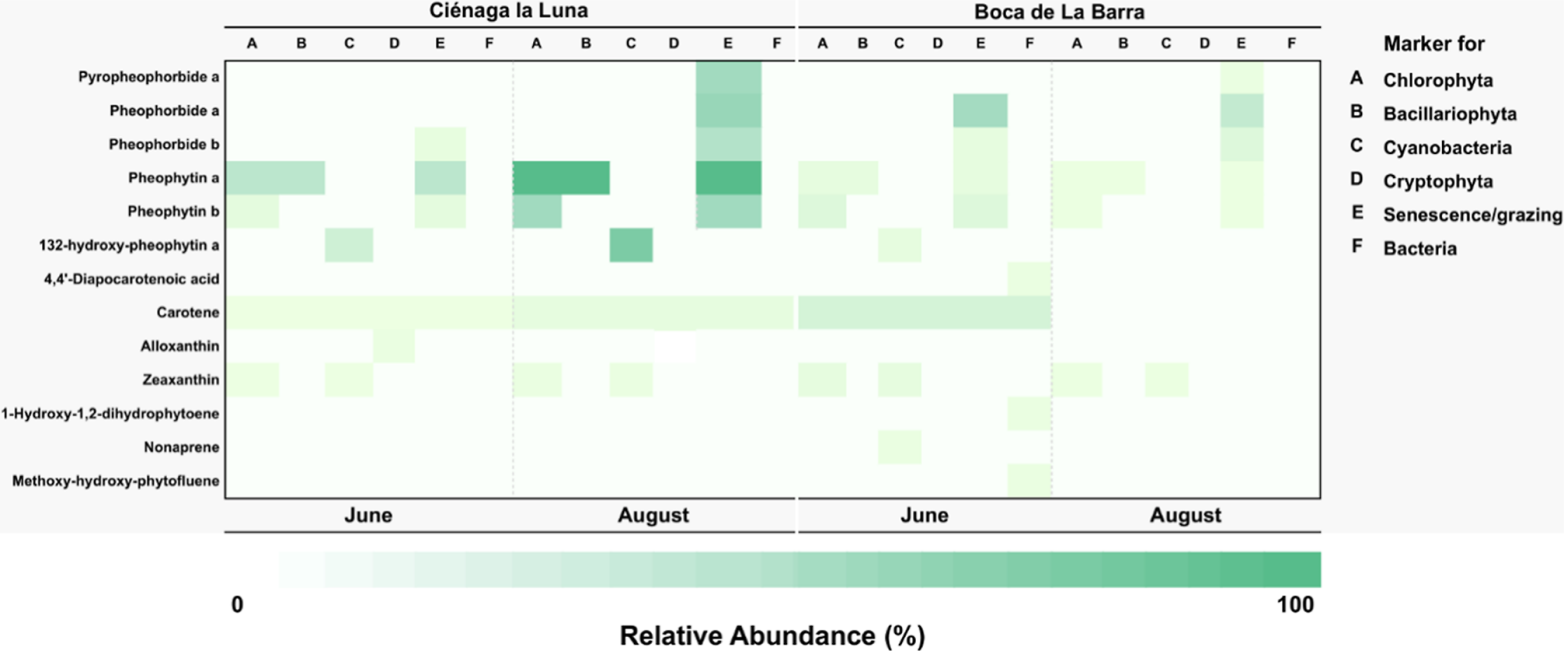
Chlorophyll derivatives and carotenoid composition in phytoplankton samples collected in June and August 2022 at Ciénaga La Luna and Boca de La Barra, CGSM, detected by MALDI FT-ICR MS. Color intensity indicates the relative abundance of each compound as a percentage. Letters denote the groups for which the respective compounds have been identified as markers. Group assignments are based on a review of scientific literature and the taxonomic ID performed in this work.

The presence of chlorophylls and carotenoids indicates photosynthetic activity in the samples, highlighting their importance in primary production and energy transfer in aquatic ecosystems.^46^ Here, chlorophyll derivatives were consistently detected as radical cations across all analyzed samples, a feature commonly associated in the literature with pigment transformation processes such as cellular senescence or grazing-related degradation in the phytoplankton community.^5,11,79^ Notably, intact chlorophyll *a*/*b* was not detected in any of the pigment extracts mass spectra, which aligns with the widely reported low survival yield of these compounds in MS.^41,80–82^ Chlorophyll degradation between sample collection and analysis could also be possible. Nevertheless, the conditions used in this work allow for the detection of intact chlorophylls in the mass spectrum, as previously reported by us.^26,34^ Additionally, the chlorophyll concentration was measured by UV–vis spectroscopy prior to MALDI-MS analysis (Table S1). Chlorophyll *a* is commonly present in phytoplankton cells, and its degradation products are diagnostic indicators of the physiological condition and grazing processes affecting the phytoplankton community.^80–82^

Here, we report the detection of radical cations of pheophytin *a* [C_55_H_74_N_4_O_5_]^+•^, pheophytin *b* [C_35_H_34_N_4_O_6_]^+•^, and pheophorbide *b* [C_35_H_34_N_4_O_6_]^+•^, at all sampling sites and months, with low mass accuracy values (∼20 ppb). Pheophytin *a*/*b* are considered markers of the phylum Chlorophyta and Bacillariophyta and senescence/grazing, and pheophorbide *a*/*b* are associated with grazing among the microorganisms composing the phytoplankton.^82^ Additionally, pyropheophorbide, [C_33_H_34_N_4_O_3_]^+•^, was detected at both sampling sites but only in the phytoplankton samples collected in August, a period of higher precipitation. These derivatives are formed during the breakdown of chlorophyll by herbivorous zooplankton or other phytoplankton microorganisms and senescent algae cells.^79,83,84^ Chlorophyll decomposition products have been used as biomarkers of organic matter derived from phytoplankton in various environments, from oligotrophic open oceans to eutrophic inland lakes.^3^ However, in shallow lagoons influenced by multiple water sources, the use of chlorophyll degradation products as biomarkers of the phytoplankton community is complicated by the presence of diverse chlorophyll inputs, including contributions from terrestrial matter, microalgae, macroalgae, and seagrasses, among others.^46^ Moreover, the biochemical and environmental mechanisms driving chlorophyll transformation in aquatic systems remain incompletely understood and continue to be the subject of ongoing research.

The presence of alloxanthin [C_40_H_52_O_2_]^+•^ in June at Ciénaga La Luna is consistent with the taxonomic identification performed, as this carotenoid is a biomarker for cryptophytes,^56^ a group previously reported in June at Ciénaga La Luna through taxonomic ID in this work. Likewise, 1-hydroxy-1,2-dihydrophytoene [C_40_H_66_O + Na]^+^ and methoxy-hydroxy-phytofluene [C_41_H_68_O_2_ + Na]^+^, detected in June in Boca de La Barra, are markers of *Rhodospirillum rubrum*,^56^ a bacterium widely distributed in aquatic environments such as ponds, lakes, streams, and standing water.^56^ Variations in terms of carotenoid molecular composition could be related to differences in light intensity, temperature, and salinity between seasons and sampling sites.^85,86^ A notable correlation was observed between the phytoplankton community structure inferred from molecular pigment analysis via MALDI-MS and that obtained through the taxonomic identification of microorganisms. Taxonomic analyses revealed the presence of representatives from Chlorophyta, Bacillariophyta, *Cyanophyta*, *Cryptophyta*, and Miozoa. In parallel, pigment profiling via MALDI FT-ICR MS enabled the detection of chlorophyll derivatives and carotenoids indicative of *Chlorophyta*, *Bacillariophyta*, *Cyanophyta*, and *Cryptophyta*, senescence and grazing processes, and the presence of bacteria. Interestingly, molecular pigment markers for Miozoa were not detected. This absence may be attributed to the relatively low abundance of peridinin (C_39_H_50_O_7_)-containing dinoflagellates at the time of sampling or possible pigment degradation. Miozoa represented between 1 and 2% of the total cell density in the samples. Furthermore, some authors have reported that peridinin may be susceptible to degradation under conditions of direct light, oxygen, and polar solvents.^87,88^ Despite this, the detection of the major phytoplankton groups, as well as bacterial-associated pigments, underscores the sensitivity and usefulness of MALDI MS for ecological assessment. The identification of bacterial pigment signatures is particularly relevant in ecosystems such as the CGSM, where high organic matter input, elevated temperatures, and eutrophic conditions foster microbial proliferation.

Another diagnostic carotenoid detected in phytoplankton samples was zeaxanthin [C_40_H_56_O_2_]^+•^, found in both June and August at Ciénaga La Luna and Boca de La Barra. This identification aligns with the taxonomic ID performed; as zeaxanthin is a well-established biomarker for the presence of cyanobacteria.^5,11,79^ As previously discussed, cyanobacteria comprised the majority of the phytoplankton community in the CGSM samples, contributing between 72 and 99% of the total cell density. A comprehensive list of all detected molecular biomarkers and their associated phytoplankton taxa and phyla is provided in Table S2, which serves as the primary reference for biomarker–taxonomic assignments discussed throughout this section. Furthermore, a comparative summary of phytoplankton groups identified by taxonomic analysis and MALDI FT-ICR MS-based biomarkers across sites and seasons is presented in Table S3. Figure 6 shows the cyanobacteria’s secondary metabolites detected in the samples collected in June and August 2022 at Ciénaga La Luna and Boca de La Barra, CGSM by MALDI FT-ICR MS.

**Figure 6:**
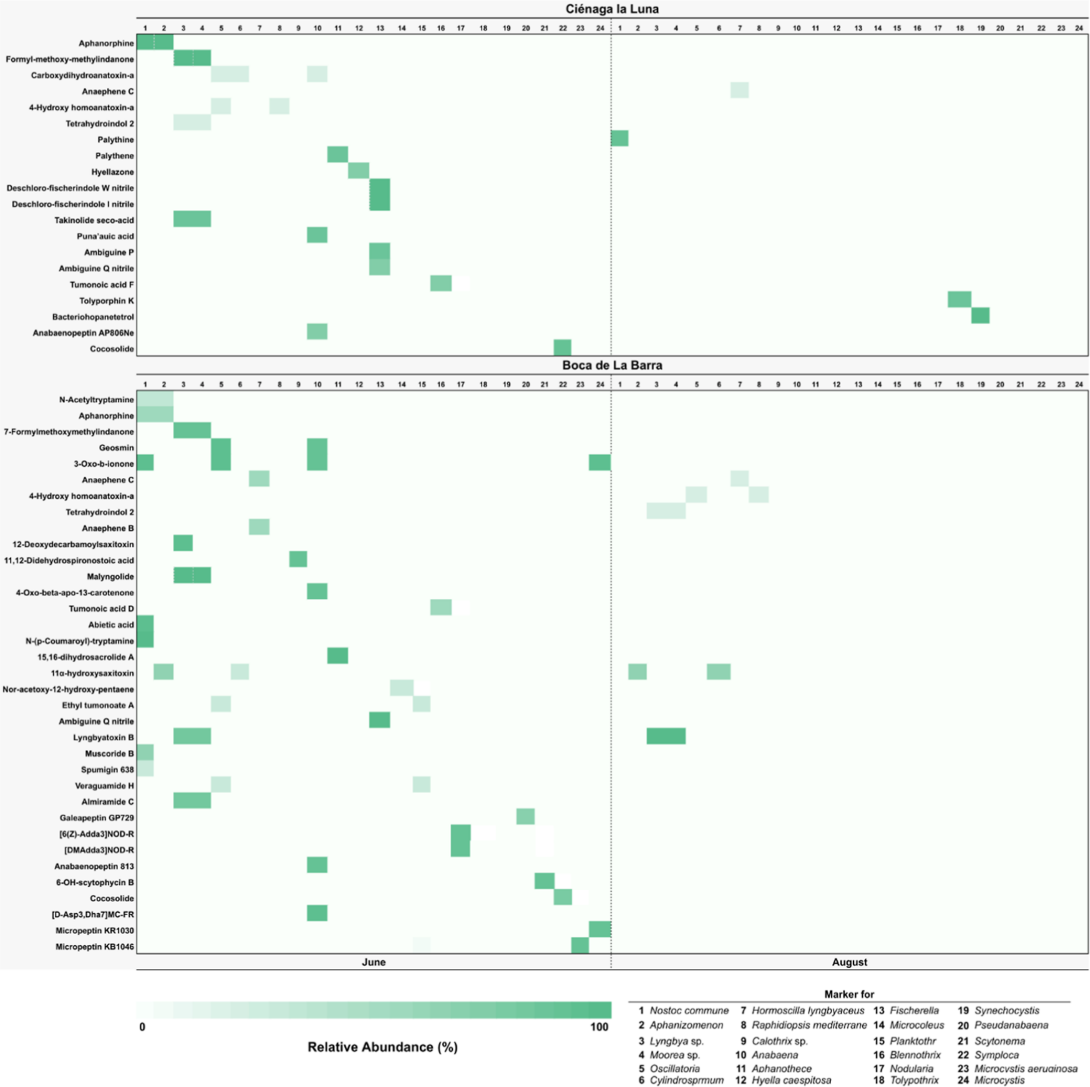
Secondary metabolites from cyanobacteria in phytoplankton samples gathered in June and August 2022 at Ciénaga La Luna and Boca de La Barra, CGSM, detected using MALDI FT-ICR MS. The color intensity shows the percentage of each compound’s relative abundance. The numbers indicate the specific cyanobacteria for which these compounds have been identified as markers.

Moreover, cyanobacteria’s secondary metabolites were the compounds with the highest molecular diversity and were identified in both months and at both sampling sites. However, June had the highest number of identified compounds in both Ciénaga La Luna and Boca de La Barra. This could be related to a greater diversity of cyanobacteria in June at both sampling sites, according to precipitation and salinity, as discussed previously. Several authors have employed secondary metabolites produced by cyanobacteria as biomarkers for the identification of cyanobacterial taxa.^57,89–91^ Owing to their molecular diversity, these compounds have also been proposed as specific biomarkers for monitoring population dynamics and responses to environmental changes in aquatic ecosystems. Among the cyanobacterial secondary metabolites identified were cyclic peptides (e.g., veraguamide H and cocosolide), cyclic nonpeptides (e.g., tolyporphin K), linear peptides (e.g., almiramide C), linear nonpeptides (e.g., aphanorphine), saxitoxins (e.g., 11α-hydroxysaxitoxin), and anabaenopeptins (e.g., anabaenopeptin AP806Ne). These compounds were detected as protonated molecules and sodium adducts, consistent with their molecular architecture and their respective proton and cation affinity profiles.^92,93^ Additionally, the detection of these adducts may be due to preformed ions in the solution and desorbed due to the sublimation properties of the matrix used (DCTB, vapor pressure 9.09 × 10^–7^ mm Hg at 25 °C).^94^

Taxonomic identification revealed the presence of potentially harmful microalgal genera in Ciénaga La Luna and Boca de La Barra, CGSM, primarily due to oceanographic conditions, anthropogenic inputs, and hydrological connectivity that promotes the transport and accumulation of phytoplankton in these shallow coastal lagoons. Interestingly, *Synechocystis*, the most abundant reported genus in this work by taxonomic ID, is a producer of bacteriohopanetetrol,^59^ detected in this work as [C_41_H_73_NO_8_]^+•^, 10 ppb, in August at Ciénaga La Luna. Bacteriohopanetetrol is a linear nonpeptide triterpenoid located in the membrane of cyanobacteria.^51^ Some authors have reported the use of bacteriohopanetetrol isomers as markers of different species, e.g., in anaerobic ammonium oxidation (anammox) in marine paleo-environments.^95^ Additionally, previously reported molecular markers associated with cyanobacterial species from the genera *Anabaena*, *Microcystis*, and *Pseudanabaena*, identified through taxonomic analysis, were also detected using MALDI FT-ICR MS. Specifically, five markers corresponding to the genus *Anabaena* were identified: 4-oxo-β-apo-13-carotenone ([C_18_H_24_O_2_]^+•^, 213 ppb), puna’auic acid ([C_18_H_32_O_4_ + Na]^+^, 168 ppb), anabaenopeptin AP806Ne ([C_41_H_58_N_8_O_9_ + Na]^+^, 51 ppb), anabaenopeptin 813 ([C_43_H_55_N_7_O_9_ + Na]^+^, 25 ppb), and [d-Asp,^3^Dha^7^]­MC-FR ([C_50_H_68_N_10_O_12_]^+•^, 89 ppb). Two compounds were associated with the genus *Microcystis*: micropeptin KR1030 ([C_53_H_74_N_8_O_13_ + Na]^+^, 127 ppb) and micropeptin KB1046 ([C_53_H_74_N_8_O_14_ + Na]^+^, 212 ppb). One marker was attributed to the genus *Pseudanabaena*: galeapeptin GP729 ([C_37_H_59_N_7_O_8_ + Na]^+^, 1 ppb).

These findings support the proposed use of medium- and low-molecular-weight biomarkers—specifically cyanobacterial secondary metabolites—in combination with chlorophylls and carotenoids as a reliable and rapid strategy for identifying phytoplankton communities in aquatic ecosystems. Interestingly, other compounds detected, such as *N*-acetyltryptamine [C_12_H_14_N_2_O + H]^+^, 3-oxo-*b*-ionone [C_13_H_18_O_2_]^+•^, palythine [C_10_H_16_N_2_O_5_ + H]^+^, muscoride B [C_31_H_41_N_5_O_6_ + H]^+^, and spumigin 638 [C_32_H_42_N_6_O_8_ + H]^+^, refer to the presence of *Nostoc commune*,^57^ widely reported cyanobacteria whose colonies grow in moist soils, on mosses and herbs, and beside streams or pools as in the CGSM. Most cyanobacteria secondary metabolites consist of species from N_n_O_o_ classes due to their cyclic peptide structures, e.g., veraguamide H (C_36_H_58_N_4_O_8_) and anabaenopeptin 813 (C_43_H_55_N_7_O_9_), detected in CGSM phytoplankton samples, serving as markers for species of the genera *Oscillatoria* ^57^ and *Anabaena*,^57^ respectively. Additionally, noncyclic peptides were assigned to N_6_O_o_ species, e.g., almiramide C (C_40_H_66_N_6_O_6_) and galeapeptin GP729 (C_37_H_59_N_7_O_8_), markers for species of the genera *Lyngbya sp*./*Moorea sp*.^57^ and *Pseudanabaena*.^57^ Additionally, seven distinct compounds indicative of cyanobacteria belonging to the genera *Lyngbya sp*. and *Moorea sp*. were detected. These microorganisms exhibit high morphological similarity but have been taxonomically differentiated based on genetic data. Both genera comprise species inhabiting tropical marine and freshwater environments and are recognized for their production of potent toxins, such as lyngbyatoxin-a, which can cause severe dermatological reactions upon skin contact.^96^ Ingestion of *Lyngbya* is potentially lethal, with most poisoning cases resulting from the consumption of fish that have bioaccumulated cyanobacteria directly or indirectly through the food web. This condition is referred to as “ciguatera-like” poisoning and is well-documented in the scientific literature.^90^

The relative abundance values of cyanobacteria’s secondary metabolites were generally low and may be related to the efficiency in the ionization of these compounds, the extraction procedure employed, or the need for a pretreatment process of the extract prior to analysis, as well as the MALDI matrix used. Here, we performed a direct MALDI-MS analysis of a mixture of phytoplankton extract; some authors have reported prior purification of the samples through Solid-Phase Extraction (SPE).^90,97^ Additionally, molecular assignments were performed by cross-referencing the molecular information generated from MALDI 21T FT-ICR MS with existing literature databases, which may be incomplete. Because of this, some assigned molecular formulas are not associated with a molecular structure in this work and would require further structural elucidation and characterization studies to confirm their identity. Additionally, the rapid and comprehensive detection of toxins present in water samples using MALDI-MS highlights the applicability of this methodology for the continuous monitoring of water quality and the ecological status of aquatic environments. This approach contributes both to the prevention of human exposure to potentially harmful substances and to the preservation of ecosystem health. Furthermore, understanding the metabolite composition based on seasonal variability can provide insights into phytoplankton community dynamics and adaptive responses to environmental changes. Figure 7 shows the molecular composition variability detected by UHR MALDI-MS across sampling seasons (June and August 2022) and sites (Ciénaga La Luna and Boca de La Barra, CGSM), analyzed through Principal Component Analysis (PCA). The first two principal components explained 61.88% and 27.09% of the total variance, respectively. This PCA reflects the molecular composition of phytoplankton samples collected at the evaluated sites and seasons. Its purpose is to assess the sensitivity of MALDI FT-ICR MS molecular fingerprints to environmentally driven and seasonal variations in phytoplankton communities rather than to serve as a predictive or population-level statistical model.

**Figure 7:**
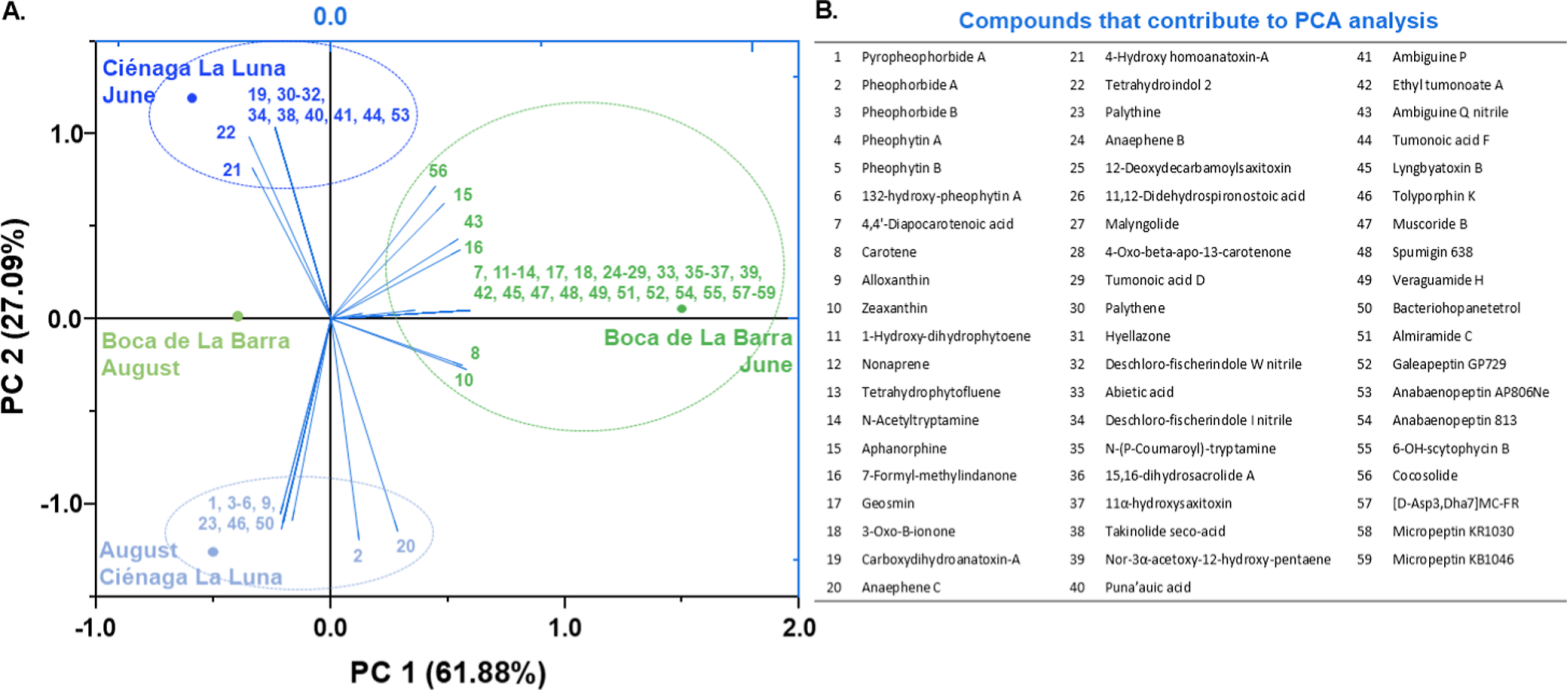
(A) PCA biplot of chlorophyll derivatives, carotenoids, and cyanobacterial metabolites identified by MALDI-FT-ICR-MS in phytoplankton samples from Ciénaga La Luna and Boca de La Barra, CGSM, during the dry (June) and rainy (August) seasons of 2022. Colors indicate sampling sites and seasons. Principal components account for 61.88% (PC1) and 27.09% (PC2) of the total variance. (B) Compounds that contribute to PCA.

The PCA conducted on the phytoplankton samples from Ciénaga La Luna and Boca de La Barra revealed that the first two principal components (PC1 and PC2) account for 88.97% of the total variance in the data set. This high percentage indicates that these two components effectively capture the majority of the variability present in the complex data set, which includes chlorophyll derivatives, carotenoids, and cyanobacterial metabolites. This approach aligns with previous studies that have successfully utilized PCA to reduce dimensionality while retaining critical data characteristics.^98^ The PCA biplot reveals a clear separation of samples according to both sampling sites and collection seasons. Samples from Ciénaga La Luna and Boca de La Barra, collected during the dry (June) and rainy (August) seasons, occupy distinct quadrants within the PCA space. This spatial distribution indicates significant variations in phytoplankton communities and their associated metabolites between the sites and seasons. Such differentiation highlights the effectiveness of rapid and direct analysis by MALDI-FT-ICR-MS in discerning ecological variations within phytoplankton assemblages, thereby validating the methodology employed in this study and supporting our previous work on the application of MALDI-MS for monitoring phytoplankton pigment profiles.^26,34,35^ As has been widely reported in the scientific literature,^43,99,100^ the observed variations in phytoplankton communities and metabolite profiles between dry and rainy seasons are related to environmental factors such as precipitation, temperature, and nutrient availability. In both samples, salinity was higher in June (dry season) than in August (rainy season), which correlates with increases in phytoplankton population density. The sample from Boca de La Barra collected in August is positioned near the origin of the PCA plot. This central positioning suggests that it does not exhibit a strong variation along the first two principal components. The blue vectors in the PCA biplot represent the influence of individual compounds on the variance observed in the data set. Notably, certain chlorophyll derivatives and cyanobacterial metabolites exhibit significant contributions, particularly in samples from Ciénaga La Luna collected in the rainy season (August), a freshwater ecosystem. These findings are consistent with previous reports indicating that rainy seasons often display lower compositional variability.^5,43,46,79^ The sample exhibiting the greatest diversity of contributions to variance was June from Boca de La Barra, aligning with the results previously discussed in this study. Interestingly, the PCA revealed that chlorophyll derivatives, such as pyropheophorbide A, pheophorbide A and B, and pheophytin A and B—although identified at all sampling sites—contributed more substantially to the variance in Ciénaga La Luna during August (the rainy season). Conversely, among the compounds that contributed most to the variance in Ciénaga La Luna in June (the dry season) were cyanobacterial metabolites. Identifying the specific compounds that contribute most significantly to each season and sampling site may allow for the proposal of these compounds as biomarkers for detecting seasonal variations associated with oceanographic conditions. This study demonstrates that the integration of pigments and cyanobacteria’s metabolite enhances the specificity of the analysis in samples containing cyanobacteria, thereby providing a robust framework for monitoring phytoplankton dynamics and identifying ecological changes.

## Conclusions

The analysis of phytoplankton samples collected from Ciénaga La Luna and Boca de La Barra during the dry (June) and rainy (August) seasons of 2022 revealed clear spatial and seasonal differences in molecular composition as resolved by ultrahigh-resolution MALDI FT-ICR MS. These differences are closely associated with environmental factors such as light intensity, temperature, salinity, and precipitation, which directly influence the structure and dynamics of phytoplankton communities. The detection of specific pigments and secondary metabolites enabled a molecular fingerprinting approach that showed strong qualitative agreement with optical-microscopy-based taxonomic identification. This consistency supports the potential of MALDI FT-ICR MS as a complementary analytical tool for phytoplankton characterization, offering substantially reduced analysis time relative to that of conventional identification methods.

Multivariate analysis using Principal Component Analysis (PCA) further highlighted distinct molecular profiles associated with seasonal and site-specific gradients, demonstrating the capability of the MALDI MS methodology to resolve chemically driven differences among phytoplankton assemblages.

The integration of pigment profiling with cyanobacterial metabolite analysis provides high analytical specificity in samples dominated by cyanobacteria, offering a robust platform that could be used to assess ecosystem health. The high sensitivity and resolution of MALDI FT-ICR MS enable rapid and accurate characterization of biomarkers of the phytoplankton communities, which could be used in the early detection of harmful algal blooms (HABs) for which additional studies would be required, incorporating broader spatial coverage, longitudinal sampling, and confirmed bloom conditions to evaluate its applicability for HAB monitoring and ecosystem health assessment.

## Supplementary Material



## Data Availability

All FT-ICR MS spectra files (.pdf) and elemental composition assignments (.xls) are publicly available via the Open Science Framework (https://osf.io/BVXAJ/) at DOI 10.17605/OSF.IO/BVXAJ in accordance with the NHFML and NSF FAIR data management plan (https://nationalmaglab.org/images/user_resources/searchable_docs/request_magnet_time/data_management_plan_policy.pdf). The corresponding authors will provide any additional reasonable requests for information.

## Notes

Environmental parameters at sampling sites (Table S1); schematic of the sampling and analytical workflow (Figure S1); mass error distribution of assigned molecular formulas (Figure S2); list of compounds detected by MALDI FT-ICR MS with detected ions, experimental *m*/*z*, mass accuracy, relative abundance, and proposed chemotaxonomic markers (Table S2); comparison between traditional taxonomy and MALDI FT-ICR MS-based chemotaxonomic markers (Table S3); isotopic and isotopic fine structure analysis at 21 T (Table S4); principal component analysis (PCA) eigenvalues and explained variance (Table S5); and supporting references (PDF)
