# Supplementary material for: Chemotaxonomic and Molecular Insights into Phytoplankton Communities in Tropical Aquatic Ecosystems via MALDI FT-ICR Mass Spectrometry
Source: ACS Meas Sci Au. 2026 Mar 24;6(3):640–54. doi: 10.1021/acsmeasuresciau.5c00175 (PMC13281183; doi:10.1021/acsmeasuresciau.5c00175)
Supplement: Supplementary file 1 [file tg5c00175_si_001.pdf]

# Chemotaxonomic and Molecular Insights into Phytoplankton Communities in Tropical Aquatic Ecosystems via MALDI FT-ICR Mass Spectrometry

*Luis M. Díaz-Sánchez*[*†,‡]; Martha L. Aguilera[§]; David Stranz[¶]; Scott Campbell[¶]; Luisa F. Espinosa-Díaz[#]; Cristian Blanco-Tirado[†]; and Marianny Y. Combariza*[*†]*

*[†]Escuela de Química, Universidad Industrial de Santander, Bucaramanga, 680002, Colombia.*

*[‡]Departamento de Química, Universidad de Pamplona, 543050 Pamplona, Colombia.*

*[§]National High Magnetic Field Laboratory, Florida State University, Tallahassee, Florida 32310, United States.*

*[¶]Sierra Analytics, Modesto, California 95356, United States.*

*[#]Instituto de Investigaciones Marinas y Costeras "José Benito Vives de Andréis"-INVEMAR, Santa Marta, Colombia.*

*Email: marianny@uis.edu.co (MYC), luis.diazsanchez@unipamplona.edu.co (LMD)

**Table S1**. Environmental parameters reported by the INVEMAR at the sampling points Ciénaga La Luna and Boca de La Barra, in June and August of 2022.[1]

| Parameter | Ciénaga La Luna | | | | | | Boca de La Barra | | | | | |
|---|---|---|---|---|---|---|---|---|---|---|---|---|
| | June | | | August | | | June | | | August | | |
| Transparency (m) | 0.4 | ± | 0.1 | 0.7 | ± | 0.1 | 1.0 | ± | 0.1 | 0.3 | ± | 0.1 |
| Salinity (g/L) | 3.0 | ± | 0.1 | 1.0 | ± | 0.1 | 9.0 | ± | 0.2 | 3.0 | ± | 0.1 |
| Temperature (˚C) | 32.0 | ± | 1.0 | 31.5 | ± | 1.0 | 28.5 | ± | 1.0 | 30.0 | ± | 1.0 |
| DO (mg $O_2$/L) | 6.1 | ± | 0.1 | 6.0 | ± | 0.1 | 6.1 | ± | 0.1 | 7.0 | ± | 0.1 |
| pH | 8.0 | ± | 0.1 | 7.8 | ± | 0.1 | 8.5 | ± | 0.1 | 8.0 | ± | 0.1 |
| Chlorophyll a (µg/L) | 48.0 | ± | 1.0 | 10.0 | ± | 1.0 | 40.0 | ± | 1.0 | 29.0 | ± | 1.0 |
| P-$PO_4$ (µg/L) | 10.0 | ± | 1.0 | 8.0 | ± | 1.0 | 3.0 | ± | 1.0 | 100.0 | ± | 1.0 |

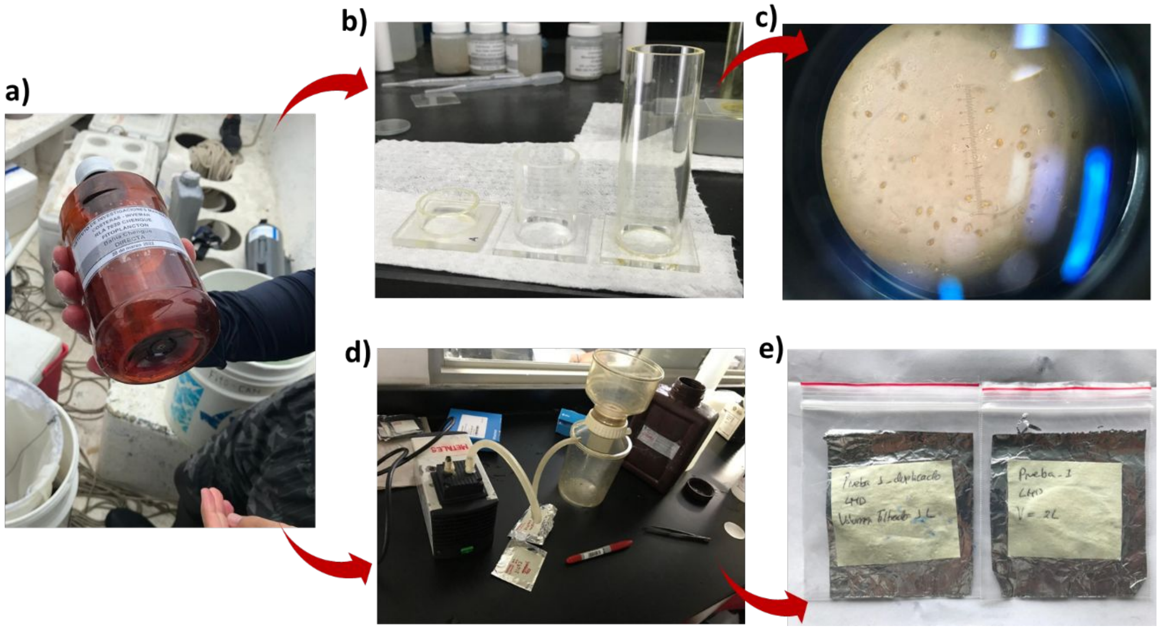


**Figure S1.** General schematic: a) Collection of phytoplankton samples in amber glass bottles at the sampling sites, subsequently divided into two portions. The first half followed the procedures for traditional taxonomic analysis: b) sedimentation of the phytoplankton samples, c) identification of phytoplankton using optical microscopy. The second half was: d) filtered, and e) stored until extraction and analysis.

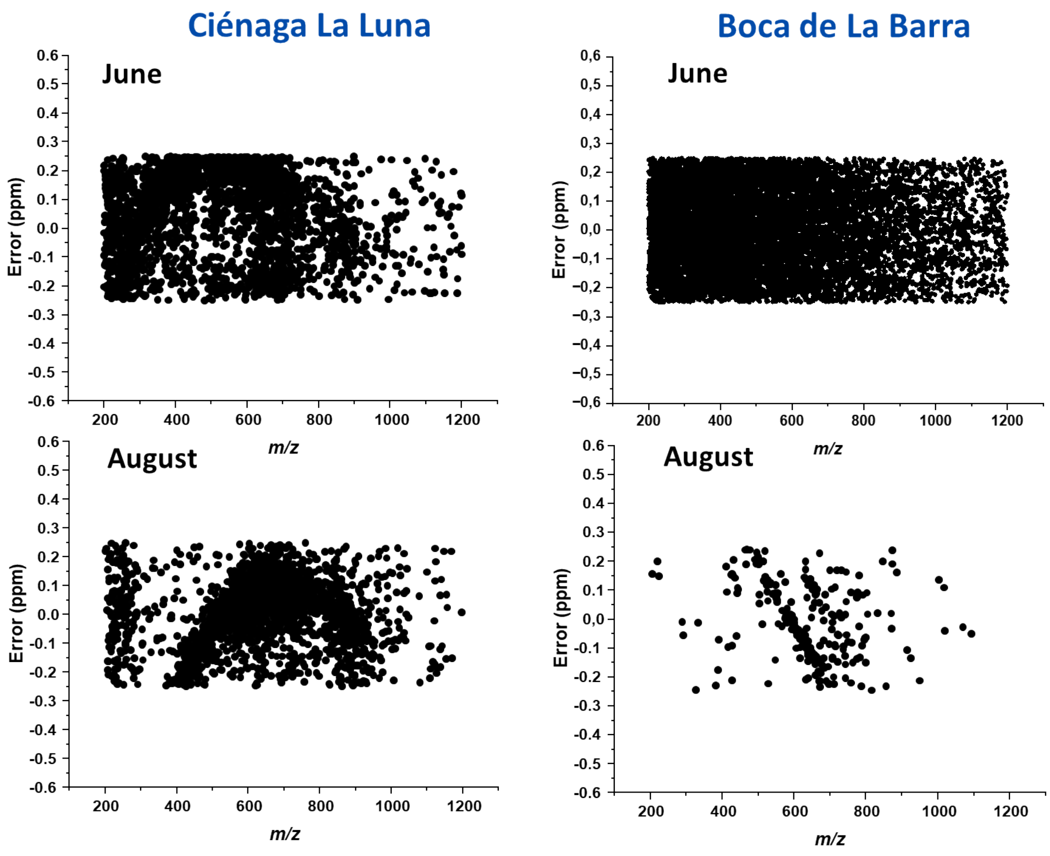


**Figure S2.** Scatter plot showing the mass error of FT-ICR-MS assigned formulas in June and August 2022 in Ciénaga La Luna and Boca de La Barra, CGSM. Mass error was calculated as the difference between the average *m/z* measured by ICR-MS and the theoretical ion mass. Assigned molecular formulas represent high-confidence elemental assignments; however, not all are linked to a unique molecular structure within the scope of this study.

**Table S2.** Compounds detected in the phytoplankton samples collected in June and August 2022 in Ciénaga La Luna and Boca de La Barra, CGSM, by MALDI FT-ICR MS.

| | | | MALDI FT-ICR MS | | | | | | | | | | | | |
|---|---|---|---|---|---|---|---|---|---|---|---|---|---|---|---|
| | | | Ciénaga La Luna | | | | | | Boca de La Barra | | | | | | |
| | | | June | | | August | | | June | | | August | | | |
| Compound[a] | | Detected ion[b] | *m/z* Exp[c] | Mass accuracy (ppb) | RA[d] (%) | *m/z* Exp[c] | Mass accuracy (ppb) | RA[d] (%) | *m/z* Exp[c] | Mass accuracy (ppb) | RA[d] (%) | *m/z* Exp[c] | Mass accuracy (ppb) | RA[d] (%) | Marker for |
| **Chlorophyll pigments** | | | | | | | | | | | | | | | |
| 1 | Pyropheophorbide a | $[C_{33}H_{34}N_4O_3]^{+•}$ | | | | 534.26253 | 18 | 18.82 | | | | 534.26261 | 125 | 0.32 | Senescence/grazing[2–4] |
| 2 | Pheophorbide a | $[C_{35}H_{36}N_4O_5]^{+•}$ | | | | 592.26804 | 40 | 29.87 | 592.26816 | 246 | 16.91 | 592.26802 | 1 | 4.99 | Senescence/grazing[5] |
| 3 | Pheophorbide b | $[C_{35}H_{34}N_4O_6]^{+•}$ | 606.24730 | 21 | 0.89 | 606.24731 | 41 | 6.84 | 606.24725 | 192 | 1.05 | 606.24727 | 32 | 1.54 | Senescence/grazing[2–4] |
| 4 | Pheophytin a | $[C_{55}H_{74}N_4O_5]^{+•}$ | 870.56537 | 1 | 5.93 | 870.56537 | 1 | 100.0 | 870.56537 | 1 | 0.91 | 870.56536 | 11 | 0.22 | Chlorophyta[2–4] Bacillariophyta[2–4] |
| 5 | Pheophytin b | $[C_{55}H_{72}N_4O_6]^{+•}$ | 884.54461 | 26 | 1.16 | 884.54462 | 9 | 19.68 | 884.54467 | 10 | 1.99 | 884.54461 | 33 | 0.23 | Chlorophyta[2–4] |
| 6 | 132-hydroxy-pheophytin a | $[C_{55}H_{74}N_4O_6]^{+•}$ | 886.56028 | 1 | 3.74 | 886.56027 | 15 | 59.92 | 886.56027 | 10 | 0.73 | | | | Cyanobacteria *Cyanobium*[2–4] |
| **Carotenoids** | | | | | | | | | | | | | | | |
| 7 | 4,4'-Diapocarotenoic acid | $[C_{30}H_{38}O_2 + Na]^+$ | | | | | | | 453.27633 | 162 | 0.33 | | | | Pseudomonadota[6] |
| 8 | Carotene | $[C_{40}H_{56}]^{+•}$ | 536.43762 | 55 | 0.21 | 536.43764 | 19 | 0.93 | 536.43767 | 17 | 2.93 | | | | Not specific[2–4] |
| 9 | Alloxanthin | $[C_{40}H_{52}O_2]^{+•}$ | | | | 564.39620 | 43 | 0.46 | | | | | | | Cryptophytes[2–4] |
| 10 | Zeaxanthin | $[C_{40}H_{56}O_2]^{+•}$ | 568.42742 | 105 | 0.23 | 568.42745 | 52 | 0.36 | 568.42743 | 83 | 0.72 | 568.42744 | 18 | 0.29 | Chlorophyta[2–4] Cyanobacteria[2–4] |
| 11 | 1-Hydroxy-1,2-dihydrophytoene | $[C_{40}H_{66}O + Na]^+$ | | | | | | | 585.50064 | 84 | 0.37 | | | | *Rhodospirillum rubrum*[6] |
| 12 | Nonaprene | $[C_{45}H_{64}]^{+•}$ | | | | | | | 604.50018 | 120 | 0.53 | | | | *Corynebacterium glutamicum*[6] |
| 13 | 1-Methoxy-1'hydroxy-1,2,1',2'-tetrahydrophytofluene | $[C_{41}H_{68}O_2 + Na]^+$ | | | | | | | 615.51122 | 102 | 0.49 | | | | *Rhodospirillum rubrum*[6] |
| **Cyanobacteria's secondary metabolites** | | | | | | | | | | | | | | | |
| 14 | N-Acetyltryptamine | $[C_{12}H_{14}N_2O + H]^+$ | | | | | | | 203.11790 | 74 | 0.34 | | | | *Nostoc commune*[5] |
| 15 | Aphanorphine | $[C_{13}H_{17}NO]^{+•}$ | 203.13041 | 188 | 0.22 | | | | 203.13047 | 31 | 0.41 | | | | *Aphanizomenon flos-aquae*[5] |

| | | | | | | | | | | | | | | | |
|---|---|---|---|---|---|---|---|---|---|---|---|---|---|---|---|
| 16 | 7-Formyl-3-methoxy-5-methylindanone | $[C_{12}H_{12}O_3]^{+\bullet}$ | 204.07804 | 207 | 0.20 | | | | 204.07805 | 214 | 0.67 | | | | *Lyngbya* sp./*Moorea* sp.[5] |
| 17 | Geosmin | $[C_{12}H_{22}O + Na]^+$ | | | | | | | 205.15625 | 188 | 0.52 | | | | *Oscillatoria*[5] |
| 18 | 3-Oxo-b-ionone | $[C_{13}H_{18}O_2]^{+\bullet}$ | | | | | | | 206.13013 | 41 | 0.52 | | | | *Nostoc commune*[5] |
| 19 | Carboxydihydroanatoxin-a | $[C_{11}H_{17}NO_3 + H]^+$ | 212.12814 | 122 | 0.21 | | | | | | | | | | *Cylindrosprmum stagnale*[5] |
| 20 | Anaephene C | $[C_{15}H_{20}O + H]^+$ | | | | 217.15865 | 200 | 0.40 | 217.15868 | 49 | 0.37 | 217.15864 | 184 | 0.22 | *Hormoscilla lyngbyaceus*[5] |
| 21 | 4-Hydroxy homoanatoxin-a | $[C_{11}H_{17}NO_2 + Na]^+$ | 218.11516 | 91 | 0.20 | | | | | | | 218.11514 | 45 | 0.24 | *Raphidiopsis mediterranea*[5] |
| 22 | Tetrahydroindol 2 | $[C_{11}H_{15}NO_4]^{+\bullet}$ | 225.09950 | 229 | 0.28 | | | | | | | 225.09954 | 88 | 0.23 | *Lyngbya*/*Moorea*[5] |
| 23 | Palythine | $[C_{10}H_{16}N_2O_5 + H]^+$ | | | | 245.11321 | 82 | 0.84 | | | | | | | *Nostoc commune*[5] |
| 24 | Anaephene B | $[C_{17}H_{22}O]^{+\bullet}$ | | | | | | | 242.16655 | 163 | 0.37 | | | | *Hormoscilla lyngbyaceus*[5] |
| 25 | 12-Deoxydecarbamoylsaxitoxin | $[C_9H_{16}N_6O + Na]^+$ | | | | | | | 247.12777 | 17 | 0.56 | | | | *Lyngbya* sp. [5] |
| 26 | 11,12-Didehydrospironostoic acid | $[C_{15}H_{20}O_3 + Na]^+$ | | | | | | | 271.13048 | 63 | 0.57 | | | | *Calothrix* sp. [5] |
| 27 | Malyngolide | $[C_{16}H_{30}O_3 + H]^+$ | | | | | | | 271.22675 | 88 | 0.82 | | | | *Lyngbya* sp./*Moorea* sp. [5] |
| 28 | 4-Oxo-beta-apo-13-carotenone | $[C_{18}H_{24}O_2]^{+\bullet}$ | | | | | | | 272.17702 | 213 | 0.59 | | | | *Anabaena*[5] |
| 29 | Tumonoic acid D | $[C_{16}H_{29}NO_3 + H]^+$ | | | | | | | 284.22206 | 154 | 0.45 | | | | *Blennothrix cantharidosmum*[5] |
| 30 | Palythene | $[C_{13}H_{20}N_2O_5 + H]^+$ | 285.14451 | 56 | 0.25 | | | | | | | | | | *Aphanothece*[5] |
| 31 | Hyellazone | $[C_{20}H_{17}NO + H]^+$ | 288.13827 | 43 | 0.25 | | | | | | | | | | *Hyella caespitosa*[5] |
| 32 | Deschloro 12-epi-fischerindole W nitrile | $[C_{21}H_{20}N_2 + H]^+$ | 301.16995 | 112 | 0.36 | | | | | | | | | | *Fischerella*[41] |
| 33 | Abietic acid | $[C_{20}H_{30}O_2]^{+\bullet}$ | | | | | | | 302.22404 | 37 | 0.58 | | | | *Nostoc commune*[5] |
| 34 | Deschloro 12-epi-fischerindole I nitrile | $[C_{21}H_{22}N_2 + H]^+$ | 303.18558 | 32 | 0.37 | | | | | | | | | | *Fischerella*[5] |
| 35 | N-(p-Coumaroyl)-tryptamine | $[C_{19}H_{18}N_2O_2 + H]^+$ | | | | | | | 307.14416 | 213 | 0.82 | | | | *Nostoc commune*[5] |
| 36 | 15,16-dihydrosacrolide A | $[C_{18}H_{30}O_4 + H]^+$ | | | | | | | 311.22167 | 45 | 0.78 | | | | *Aphanothece*[5] |
| 37 | 11α-hydroxysaxitoxin | $[C_{10}H_{17}N_7O_5]^{+\bullet}$ | | | | | | | 315.12862 | 186 | 0.37 | 315.12914 | 63 | 0.39 | *Aphanizomenon flos-aquae*[41] |
| 38 | Takinolide seco-acid | $[C_{17}H_{34}O_4 + Na]^+$ | 325.23496 | 119 | 0.26 | | | | | | | | | | *Lyngbya sp./Moorea sp.*[5] |
| 39 | 20-Nor-3α-acetoxy-12-hydroxy-abieta-5,7,9,11,13-pentaene | $[C_{21}H_{26}O_3]^{+\bullet}$ | | | | | | | 326.18767 | 86 | 0.49 | | | | *Microcoleus*[5] |

| | | | | | | | | | | | | | | | |
|---|---|---|---|---|---|---|---|---|---|---|---|---|---|---|---|
| 40 | Puna'auic acid | $[C_{18}H_{32}O_4 + Na]^+$ | 335.21933 | 168 | 0.24 | | | | | | | | | | *Anabaena*[5] |
| 41 | Ambiguine P | $[C_{25}H_{29}NO + H]^+$ | 360.23224 | 156 | 0.27 | | | | | | | | | | *Fischerella ambigua*[5] |
| 42 | Ethyl tumonoate A | $[C_{21}H_{37}NO_4]^{+\bullet}$ | | | | | | | 367.27167 | 99 | 0.30 | | | | *Oscillatoria/Planktothr*[5] |
| 43 | Ambiguine Q nitrile | $[C_{26}H_{28}N_2]^{+\bullet}$ | 368.22477 | 202 | 0.24 | | | | 368.22477 | 214 | 0.69 | | | | *Fischerella ambigua*[5] |
| 44 | Tumonoic acid F | $[C_{21}H_{37}NO_5 + H]^+$ | 384.27453 | 214 | 0.22 | | | | | | | | | | *Blennothrix*[5] |
| 45 | Lyngbyatoxin B | $[C_{27}H_{39}N_3O_3 + Na]^+$ | | | | | | | 476.28834 | 36 | 0.41 | 476.28892 | 20 | 0.85 | *Lyngbya sp./Moorea sp.*[5] |
| 46 | Tolyporphin K | $[C_{30}H_{32}N_4O_4]^{+\bullet}$ | | | | 512.24180 | 47 | 0.60 | | | | | | | *Tolypothrix*[5] |
| 47 | Muscoride B | $[C_{31}H_{41}N_5O_6 + H]^+$ | | | | | | | 580.31297 | 18 | 0.42 | | | | *Nostoc commune*[5] |
| 48 | Spumigin 638 | $[C_{32}H_{42}N_6O_8 + H]^+$ | | | | | | | 639.31354 | 228 | 0.32 | | | | *Nostoc commune*[5] |
| 49 | Veraguamide H | $[C_{36}H_{58}N_4O_8]^{+\bullet}$ | | | | | | | 674.42486 | 73 | 0.30 | | | | *Oscillatoria/Planktothrix*[5] |
| 50 | Bacteriohopanetetrol | $[C_{41}H_{73}NO_8]^{+\bullet}$ | | | | 707.53299 | 10 | 1.25 | | | | | | | *Synechocystis*[5] |
| 51 | Almiramide C | $[C_{40}H_{66}N_6O_6 + H]^+$ | | | | | | | 727.51161 | 65 | 0.48 | | | | *Lyngbya sp./Moorea sp.*[5] |
| 52 | Galeapeptin GP729 | $[C_{37}H_{59}N_7O_8 + Na]^+$ | | | | | | | 752.43173 | 1 | 0.33 | | | | *Pseudanabaena*[5] |
| 53 | [6(Z)-Adda[3]]NOD-R | $[C_{41}H_{60}N_8O_{10}]^{+\bullet}$ | | | | | | | 824.44262 | 86 | 0.39 | | | | *Nodularia*[5] |
| 54 | Anabaenopeptin AP806Ne | $[C_{41}H_{58}N_8O_9 + Na]^+$ | 829.42193 | 51 | 0.22 | | | | | | | | | | *Anabaena*[5] |
| 55 | [DMAdda[3]]NOD-R | $[C_{40}H_{58}N_8O_{10} + Na]^+$ | | | | | | | 833.41682 | 22 | 0.38 | | | | *Nodularia*[5] |
| 56 | Anabaenopeptin 813 | $[C_{43}H_{55}N_7O_9 + Na]^+$ | | | | | | | 836.39536 | 25 | 0.44 | | | | *Anabaena*[5] |
| 57 | 6-OH-scytophycin B | $[C_{45}H_{73}NO_{13} + H]^+$ | | | | | | | 836.51548 | 22 | 0.41 | | | | *Scytonema*[5] |
| 58 | Cocosolide | $[C_{46}H_{76}O_{16}]^{+\bullet}$ | 884.51277 | 22 | 0.29 | | | | 884.51275 | 45 | 0.45 | | | | *Symploca*[5] |
| 59 | [D-Asp[3],Dha[7]]MC-FR | $[C_{50}H_{68}N_{10}O_{12}]^{+\bullet}$ | | | | | | | 1000.50118 | 89 | 0.57 | | | | *Anabaena*[5] |
| 60 | Micropeptin KR1030 | $[C_{53}H_{74}N_8O_{13} + Na]^+$ | | | | | | | 1053.52662 | 127 | 0.43 | | | | *Microcystis aeruginosa*[5] |
| 61 | Micropeptin KB1046 | $[C_{53}H_{74}N_8O_{14} + Na]^+$ | | | | | | | 1069.52189 | 212 | 0.47 | | | | *Microcystis*[5] |

[a]Some formulas listed herein are not linked to a unique molecular structure in this work. The assigned compounds may have constitutional isomers that could not be distinguished in this study.
[b]Detected ion.
Radical cation: molecular ion formed by the removal of one electron, resulting in a positively charged species with an unpaired electron in its electronic ground state, $M^{+\bullet}$.
Protonated molecule: ion generated by proton attachment to the neutral molecule, represented as $[M + H]^+$.
Sodium adduct: ion formed by non-covalent association of a sodium cation with the neutral molecule, represented as $[M + Na]^+$.
[c]*m/z* Exp: experimental mass-to-charge ratio.
[d]RA: relative abundance, defined as the intensity of a given ion normalized to the total ion intensity within the same mass spectrum.

**Table S3.** Comparative overview of phytoplankton groups identified by traditional taxonomic analysis and MALDI FT-ICR MS–based chemotaxonomic markers across sampling sites and seasons.*

| | Ciénaga La Luna | | | | Boca de la Barra | | | |
|---|---|---|---|---|---|---|---|---|
| | June | | August | | June | | August | |
| Phytoplankton group | Taxonomic ID | MALDI FT-ICR | Taxonomic ID | MALDI FT-ICR | Taxonomic ID | MALDI FT-ICR | Taxonomic ID | MALDI FT-ICR |
| Chlorophyta | ✓ | ✓ | ✓ | ✓ | ✓ | ✓ | ✓ | ✓ |
| Cyanobacteria | ✓ | ✓ | ✓ | ✓ | ✓ | ✓ | ✓ | ✓ |
| Bacillariophyta | ✓ | ✓ | – | – | – | – | ✓ | ✓ |
| Cryptophyta | ✓ | ✓ | – | ✓ | – | – | – | – |
| Miozoa | ✓ | – | – | – | – | – | ✓ | – |
| Cell senescence / grazing | – | ✓ | – | ✓ | – | ✓ | – | ✓ |
| Bacteria** | – | ✓ | – | – | – | ✓ | – | – |

* Indicates detection of diagnostic taxonomic features (microscopy-based identification) or characteristic molecular biomarkers identified by MALDI FT-ICR MS (see Table S2). Empty cells indicate that the corresponding group was not confidently identified by that specific approach under the applied analytical criteria. Detection by MALDI FT-ICR MS is based on chemotaxonomic markers and does not imply quantitative abundance.

** Bacteria (non-phytoplankton). Bacterial metabolites and non-algal signals detected.

**Table S4.** Comparison of Isotopic and Isotopic Fine Structure of some of compounds detected as $M^{+\bullet}$ in June and August 2022 in Ciénaga La Luna and Boca de La Barra, CGSM, by 21 Tesla MALDI FT-ICR MS, providing a mass resolving power of 1.3x$10^6$ at *m/z* 400.

| **Compound** | **Molecular formula** | **Isotopic pattern** | |
|---|---|---|---|
| | | **Theoretical*** | **Experimental** |
| Pheophytin a | $C_{55}H_{74}N_4O_5$ | 100:1.46: 59.68:0.85:0.87 1.04:17.48:0.51:0.26: 3.36:0.15 | 100:1.46: 59.68:0.85:0.87 1.04:17.48:0.51:0.26: 3.36 |
| Carotene | $C_{40}H_{56}$ | 100:43.26: 0.64:9.12:0.28 1.25:0.13 | 100:43.26: 0.64:9.12:0.28 1.25 |
| Lyngbyatoxin B | $C_{27}H_{39}N_3O_3$ | 100:1.10: 29.32:0.45:0.32 0.62:4.14:0.13:0.18:0.38 | 100:1.10: 29.32:0.45 0.62:4.14:0.38 |
| Bacteriohopanetetrol | $C_{41}H_{73}NO_8$ | 100:0.37 44.65:0.84:0.16 1.65:9.73:0.37:0.73:1.39 | 100:0.37 44.65:0.84 1.65:9.73:0.73:1.39 |

*Theoretical Isotopic pattern was calculated for the same resolving power using the ChemCal molecular formula calculator algorithm.

**Table S5.** Principal Component Analysis (PCA): Eigenvalues, Explained Variance, and Variable Cumulative of the FT-ICR MS data analysis of phytoplankton samples collected in June and August 2022 in Ciénaga La Luna and Boca de La Barra, CGSM.

| PC | Eigenvalue | Percentage of Variance | Cumulative |
|---|---|---|---|
| 1 | 36,50958 | 61.88% | 61.88% |
| 2 | 15,98263 | 27.09% | 88.97% |
| 3 | 6,5078 | 11.03% | 100.00% |
| 4 | 0 | 0.00% | 100.00% |

References

(1) INVEMAR. *Monitoreo de Las Condiciones Ambientales y Los Cambios Estructurales y Funcionales de Las Comunidades Vegetales y de Los Recursos Pesqueros Durante La Rehabilitación de La Ciénaga Grande de Santa Marta*; 2022.

(2) Roy, S.; Llewellyn, C.; Skarstad, E.; Johnsen, G. *Phytoplankton Pigments: Characterization, Chemotaxonomy and Applications in Oceanography*; 2011.

(3) Wright, S.; Jeffrey, S. Pigment Markers for Phytoplankton Production. *Hdb. Env. Chem.* **2006**, *2*, 71–104.

(4) Jeffrey, S.; Mantoura, R.; Wright, S. *Phytoplankton Pigments in Oceanography: Guidelines to Modern Methods*, Primera.; Paris, 1997; Vol. 48.

(5) Jones, M. R.; Pinto, E.; Torres, M. A.; Dörr, F.; Mazur-Marzec, H.; Szubert, K.; Tartaglione, L.; Dell'Aversano, C.; Miles, C. O.; Beach, D. G.; McCarron, P.; Sivonen, K.; Fewer, D. P.; Jokela, J.; Janssen, E. M. L. CyanoMetDB, a Comprehensive Public Database of Secondary Metabolites from Cyanobacteria. *Water Res* **2021**, *196*. https://doi.org/10.1016/j.watres.2021.117017.

(6) *Carotenoids Database, available at: http://carotenoiddb.jp Consultation date: May 7, 2024.*
